# Pharmacological rescue of cognitive function in a mouse model of chemobrain

**DOI:** 10.1186/s13024-021-00463-2

**Published:** 2021-06-26

**Authors:** Lien D. Nguyen, Tom T. Fischer, Barbara E. Ehrlich

**Affiliations:** 1 Department of Pharmacology, Yale University, New Haven, CT 06520 USA; 2 Interdepartmental Neuroscience Program, Yale University, New Haven, CT 06520 USA; 3 Present Address: Department of Neurology, Ann Romney Center for Neurologic Diseases, Brigham and Women’s Hospital and Harvard Medical School, Boston, MA 02115 USA; 4 Institute of Pharmacology, University of Heidelberg, Heidelberg, Germany

**Keywords:** Calcium, Paclitaxel, Protein kinase C, Dendrites, Spines

## Abstract

**Background:**

After chemotherapy, many cancer survivors suffer from long-lasting cognitive impairment, colloquially known as “chemobrain.” However, the trajectories of cognitive changes and the underlying mechanisms remain unclear. We previously established paclitaxel-induced inositol trisphosphate receptor (InsP3R)-dependent calcium oscillations as a mechanism for peripheral neuropathy, which was prevented by lithium pretreatment. Here, we investigated if a similar mechanism also underlay paclitaxel-induced chemobrain.

**Method:**

Mice were injected with 4 doses of 20 mg/kg paclitaxel every other day to induced cognitive impairment. Memory acquisition was assessed with the displaced object recognition test. The morphology of neurons in the prefrontal cortex and the hippocampus was analyzed using Golgi-Cox staining, followed by Sholl analyses. Changes in protein expression were measured by Western blot.

**Results:**

Mice receiving paclitaxel showed impaired short-term spatial memory acquisition both acutely 5 days post injection and chronically 23 days post injection. Dendritic length and complexity were reduced in the hippocampus and the prefrontal cortex after paclitaxel injection. Concurrently, the expression of protein kinase C α (PKCα), an effector in the InsP3R pathway, was increased. Treatment with lithium before or shortly after paclitaxel injection rescued the behavioral, cellular, and molecular deficits observed. Similarly, memory and morphological deficits could be rescued by pretreatment with chelerythrine, a PKC inhibitor.

**Conclusion:**

We establish the InsP3R calcium pathway and impaired neuronal morphology as mechanisms for paclitaxel-induced cognitive impairment. Our findings suggest lithium and PKC inhibitors as candidate agents for preventing chemotherapy-induced cognitive impairment.

**Supplementary Information:**

The online version contains supplementary material available at 10.1186/s13024-021-00463-2.

## Background

The number of cancer survivors has increased rapidly due to improvements in awareness, screening, prevention, diagnosis, and treatment [1]. Yet, cancer treatments are associated with severe, long-lasting, and sometimes irreversible side effects. Recent evidence from structural [2, 3] and functional [4–6] imaging studies on cancer survivors shows that chemotherapy-induced cognitive impairment, or “chemobrain,” affects between 17 and 75% of cancer survivors [7], some many years after treatment ends. Symptoms of chemobrain include memory lapses, learning difficulties, and troubles with focusing, planning, and multitasking [8–10]. With an estimated 16 million cancer survivors in the US alone [11], preventing or alleviating chemobrain is an urgent clinical need. Because the onset of the neurological insult, which is the start of chemotherapy, is known, the initiation phase of chemobrain is a promising timepoint for intervention. Determining the cellular and molecular mechanisms of chemobrain will also facilitate the discovery of better prevention and treatment options.

Here, we focus on paclitaxel, which is often the first-line treatment for prevalent cancer types, including breast cancer, ovarian cancer [12–14], and other solid cancers [15, 16]. The antitumor effect of paclitaxel is attributed to the stabilization of tubulin polymers [17], causing mitotic arrest and apoptosis [18]. However, paclitaxel is responsible for numerous side effects that appear to be tubulin-independent, including peripheral neuropathy [19]. We previously elucidated a mechanism for paclitaxel-induced peripheral neuropathy, in which paclitaxel binds neuronal calcium sensor 1 (NCS1) to induce spontaneous InsP3R-dependent calcium oscillations [20–27]. Through blocking calcium oscillations [21], lithium pretreatment rescued paclitaxel-induced peripheral neuropathy in a mouse model [26]. Lithium is a clinically approved drug for treating depression and bipolar disorders since the 1950s [28], and has been shown to be beneficial in animal models of TBI, aging, Alzheimer’s disease (AD), and other neurodegenerative diseases [29]. Recent studies found that paclitaxel and its analog docetaxel can penetrate the blood-brain barrier and accumulate in the central nervous system (CNS) [30–32]. Furthermore, dysregulated calcium release via the InsP3R has been implicated in cognitive impairment in AD [33] and psychological stress [34]. Therefore, we aimed to further investigate the effect of paclitaxel in the CNS. We hypothesized that the mechanism and successful treatment with lithium we observed for paclitaxel-induced peripheral neuropathy would also apply to cognitive impairment.

In this study, we established a mouse model of chemobrain in which 4 injections of 20 mg/kg of paclitaxel impaired short-term spatial memory acquisition in mice both acutely at 5 days post-injection (DPI) and chronically at 23 DPI. Using Golgi-Cox staining, we observed altered neuronal morphology in the dentate gyrus and the frontal cortex. We also found an upregulation of protein kinase C α (PKCα), an effector in the InsP3R signaling pathway, acutely in the cortex and hippocampus, and chronically in the cortex. Pretreatment with lithium or the PKC inhibitor chelerythrine rescued deficits induced by paclitaxel injections. Additionally, posttreatment with lithium up to 10 days after paclitaxel injection reversed the memory deficits, but not when administered later, suggesting a limited time window for rescuing chemobrain. Overall, we provide evidence that dysregulation in the InsP3R calcium signaling pathway and disruption of neuronal morphology contribute to paclitaxel-induced cognitive impairment, and that targeting this pathway is a promising approach to prevent chemobrain.

## Materials and methods

### Animal use and treatment

This study was carried out in accordance with the recommendations in the U.S. National Institutes of Health Guide for the Care and Use of Laboratory Animals. The protocol was approved by the Institutional Animal Care and Use Committee at Yale University, and all efforts were made to minimize suffering. Mice were maintained on a 12:12-h light/dark cycle (7:00 am on/7:00 pm off) with food and water provided ad libitum before experimental procedures. All animal experiments were performed during the light cycle.

### Paclitaxel-induced model of cognitive impairment

Seven-week-old female C57BL/6 mice were purchased from Charles River and allowed to habituate to the facility for 7 days, followed by 3 days of handling before the start of the experiment. Mice were randomly assigned into groups, with all groups represented in each cage. Depending on the treatment group, lithium chloride (Sigma-Aldrich, 12.8 mg/kg in 0.9% saline), chelerythrine chloride (Cayman Chemical, 2 mg/kg in 0.4% DMSO in saline), or the appropriate vehicle was administered intraperitoneally 1 h before injection of vehicle (20% 50:50 Cremophor EL: ethanol, 80% saline) or paclitaxel (Cayman Chemical, 20 mg/kg in 20% 50:50 Cremophor EL: ethanol, 80% saline) to induce cognitive impairment. Each mouse received a total of 4 pairs of injections over 8 days. During and after injections, mice were weighed daily and checked for general health and any sign of pain or distress.

### Open-field exploration and displaced object recognition

Behavioral experiments were carried out as previously described [35, 36]. Data were analyzed blinded to the experimental conditions. Open-field exploration (OFE) and displaced object recognition (DOR) tasks were carried out over two consecutive days. The experimental arena was a 35x70x35 cm opaque, white Plexiglas chamber. The arena was covered with ~ 1 cm of standard corn cob bedding. After each mouse, feces were removed, and the bedding was shaken to distribute odor cues equally. A camera was mounted 100 cm above the arena to record the test sessions. The test was conducted during the mice’s light phase under low light condition (45 Lux). 1 h before testing, mice were brought into the testing room and allowed to habituate to the room.

For the OFE task, each mouse was allowed to explore the arena for 10 min. The camera footage was then analyzed using ToxTrac, a published program for the total distance moved and time spent in the peripheral versus the central areas [37]. For the DOR task, pairs of 50-mL Falcon tubes filled with corn cob bedding were taped cap-down to pre-determined positions in the arena. They were selected specifically because mice were unable to climb onto the pointed ends of the tubes. During the familiarization phase, each mouse was first allowed to explore the arena where the two Falcon tubes were placed in symmetrical locations for 5 min before being taken out and returned to its home cage. 2 h later, the mouse was returned to the arena for another 5 min, with 1 tube remaining in the same position and 1 tube moved to a different position. The positions of the tubes were counterbalanced. After each mouse, the tubes were sprayed with water and wiped dry to remove odor cues. The camera footage was then analyzed for bouts of interactions with the tubes. Sniffing and biting were considered to be interactions. Casual touching of the tubes in passing or leaning onto the tubes to look around was not counted. The displacement object’s preference index was calculated as 100* (time spent with displaced object) / (total time spent with both displaced and familiar objects). The preference index for the familiar object was similarly calculated.

### Euthanasia and tissue collection

Golgi-Cox staining solutions were prepared in advance, according to a published protocol [38]. For tissue collection, each mouse was first anesthetized for ~ 30 s with 30% isoflurane and then quickly decapitated with scissors. The skull was opened, and the brain was extracted and washed briefly in ice-cold 1X phosphate-buffered saline solution (PBS, AmericanBio). A razor blade was then used to dissect the brain into two hemispheres along the medial longitudinal fissure. One hemisphere was immediately dropped into a 25-mL scintillation vial containing 10 mL of impregnation stock solution. The other hemisphere was rapidly dissected into the hippocampus and the frontal cortex, snap-frozen in liquid nitrogen, and then stored at -80 °C until further use.

### Golgi-Cox staining, imaging, and quantification

Golgi-Cox staining was performed according to a published protocol [38]. Briefly, the samples were impregnated with a potassium dichromate and mercuric chloride solution at room temperature for 7 days, then immersed in a cryoprotection solution for 4 days, and then sectioned into 200 μm frontal slices with a vibratome. The Golgi Atlas of the Postnatal Mouse Brain was used as the reference to identify the section position of the slices [39]. Slices corresponding to frontal sections 10 and 11 in the atlas were selected for imaging the hippocampus and the parietal cortex. Slices corresponding to frontal sections 4 and 5 were selected for imaging the prefrontal cortex. The selected slices were mounted on 0.3% gelatin-coated slides, developed and dehydrated through a series of increasing alcohol concentrations, then with xylene, and finally mounted in Eukitt solution (Sigma Aldrich) for imaging. Z-stack images of different regions, including the dentate gyrus and the frontal cortex, were collected using a Zeiss LSM 710 Duo microscope with 20X and 60X objectives. Neurons that showed intact and complete dendritic arbors, consistent dark staining, and relative isolation to other neurons were selected for imaging. Spines were imaged from basal dendritic branches at least 50 μm away, and apical dendritic branches at least 100 μm away, from the cell soma. Scholl analysis, total dendritic length, number of branch points, and spine density were performed with ImageJ using the Simple Neurite Tracer plugin [40]. Spine density was quantified as the number of protrusions on dendritic branches per μm dendritic length.

### Tissue lysis and Western blot

Frozen tissues were thawed in RIPA buffer containing protease inhibitor, phenylmethylsulfonyl fluoride (PMSF), and sodium orthovanadate (Santa Cruz), homogenized with a polytron, and then spun down twice at 13000 rpm, 4 °C to remove cell debris. Protein concentration was quantified using Pierce BCA protein assay kit (ThermoFisher Scientific) according to the manufacturer’s instruction. Western blots were performed using the NuPAGE system (ThermoFisher Scientific) and PVDF membrane with the Biorad wet transfer system (Bio-Rad Laboratories). Approximately 20 μg total protein was loaded into each lane. Information about the antibodies used is included in Supp. Table 1.

### Statistical analyses

Data management and calculations were performed using PRISM Statistical Software 8 (GraphPad Software). The specific statistic tests were detailed in the figure legend and Supp. Table 2. Generally, two-tailed unpaired student t-tests were used to compare two groups. One-way analysis of variance (ANOVA) followed by Tukey’s post-hoc tests were used to compare multiple groups. Two-way repeated ANOVA followed by Dennet’s post-hoc tests were used for Sholl analyses. A *p*-value < 0.05 was considered to be statistically significant and the following notations were used in all figures: * for *p* < 0.05, ** for *p* < 0.01, *** for *p* < 0.001, and **** for *p* < 0.0001. For Sholl analysis graphs, error bars shown were standard error of the mean (SEM). For all other graphs, error bars shown were standard deviation (SD).

## Results

### Establishing a mouse model of chemobrain

**Fig. 1:**
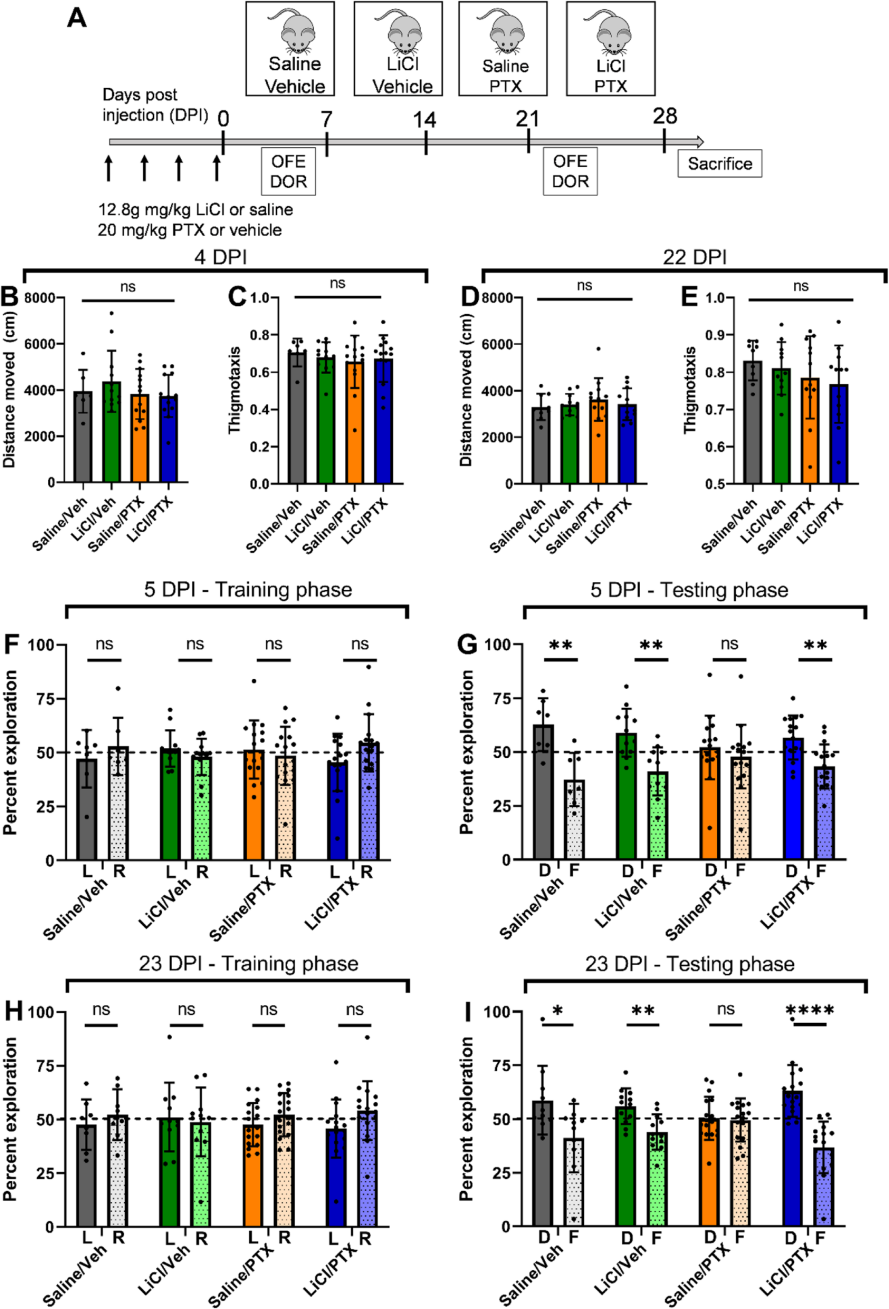
Lithium pretreatment rescues paclitaxel-induced short-term memory impairment. (A) Schematic illustration of paclitaxel and lithium injection scheme, followed by behavioral tasks (OFE = open-field exploration, DOR = displaced object recognition, D = displaced, F = familiar). (B-E) Locomotor activity and anxiety were similar across all groups at 4 and 22 DPI. One-way ANOVA, *p* > 0.3 for all comparisons. (F-I) Paclitaxel treatment impaired short-term memory acquisition, which was rescued by lithium pre-treatment (t-test, adjusted for multiple comparisons). 5 DPI: (F) *p* > 0.2 for all groups (G) *p* = 0.004 for Saline/Veh, *p* = 0.003 for LiCl/Veh, *p* = 0.41 for Saline/PTX, p = 0.003 for LiCl/PTX. 23 DPI: (H) p > 0.3 for all groups. (I) *p* = 0.047 for Saline/Veh, *p* = 0.005 for LiCl/Veh, *p* = 0.70 for Saline/PTX, *p* < 0.0001 for LiCl/PTX. *N* = 8–17 mice per group

To measure cognitive function, we selected the displaced object recognition task (DOR) task with a 2h interval between the familiarization and the test sessions. Optimal performance on this task requires contribution from both the hippocampus and the cortex, as either prefrontal cortical or hippocampal lesions were sufficient to impair task performance [41]. In addition, the short inter-session interval also puts greater emphasis on cortex-dependent working and short-term memory instead of long-term memory consolidation in the hippocampus [42]. Here, we utilized female mice because paclitaxel is a major treatment for breast, lung, and ovarian cancers, which are among the most common cancer types in women [43]. Notably, previous studies of taxane CNS toxicity used only male mice [44]. After optimizing dose and injection schemes, we established that 4 intraperitoneal (IP) injections of 20 mg/kg paclitaxel were sufficient to impair DOR task performance (Fig. 1 & Supp. Fig. 1). No significant differences in weight loss were observed among the groups (Supp. Fig. 1B). 4 × 20 mg/kg paclitaxel translates to 4 × 60 = 240 mg/m^2^ in humans [45]. In one commonly used chemotherapeutic protocol, cancer patients receive 1 intravenous (IV) infusion of 180 mg/m^2^, repeated every 3 weeks for 6 cycles [46]. Although IP injections and IV infusions are not equivalent, our injection regimen is roughly comparable to 1 standard treatment cycle and well below the total accumulated amount. A similar dosage was shown to effectively inhibit tumor growth in xenograft models of breast [26, 47] and liver cancers [48]. We next optimized the dose of lithium in our model and found that pretreatment with 12.8 mg/kg LiCl before each PTX injection rescued the performance in the DOR task, both at 5 DPI and 23 DPI (Supp. Fig. 1C). This dose resulted in a peak plasma lithium level of 0.36 mM (Supp. Fig. 2), which is below the lower therapeutic target range (0.5 to 0.8 mM) in humans [49].

### Lithium pretreatment rescues paclitaxel-induced short-term memory impairment

Mice were randomly divided into 4 groups, with each group receiving either saline or 12.8 mg/kg lithium, and then 1 h later, either vehicle or 20 mg/kg paclitaxel. Mice received a total of 4 pairs of injections, 1 pair every two days. No significant weight loss was observed in all groups over the injection duration (Supp. Fig. 3). Mice were tested with the open-field exploration (OFE) and the DOR tasks at 4 and 5 DPI, respectively, to measure the acute effects of paclitaxel toxicity. Both tests were then repeated at 22 and 23 DPI to measure the chronic effects (Fig. 1A). The OFE task measured locomotor performance through the total distance traveled and anxiety through the thigmotaxis index. A higher thigmotaxis index indicates higher levels of anxiety [50]. We observed no differences among the groups (Fig. 1B-E), suggesting that paclitaxel neither impaired locomotor activity nor caused increased anxiety.

Short-term memory impairment was measured using the DOR task. At 5 DPI (Fig. 1F), during the training phase, mice showed no preference for the left or the right object (Fig. 1H). During the testing phase (Fig. 1I), control mice receiving only vehicle injection with saline or lithium showed a significant preference for the displaced object. In contrast, mice receiving paclitaxel with saline showed no preference for either object, suggesting that they had impaired short-term memory acquisition. Similar to what we previously reported for the peripheral nervous system [26], lithium pretreatment also rescued the preference for the displaced object. Total interaction time was similar among all groups, suggesting that this was not a confounding factor (Supp. Fig. 4). The memory deficits in mice receiving paclitaxel persisted up to 23 DPI (Fig. 1H-I), suggesting long-lasting impairment, which could similarly be prevented by lithium pretreatment.

### Within a limited time window, lithium treatment after paclitaxel reverses memory impairment

**Fig. 2:**
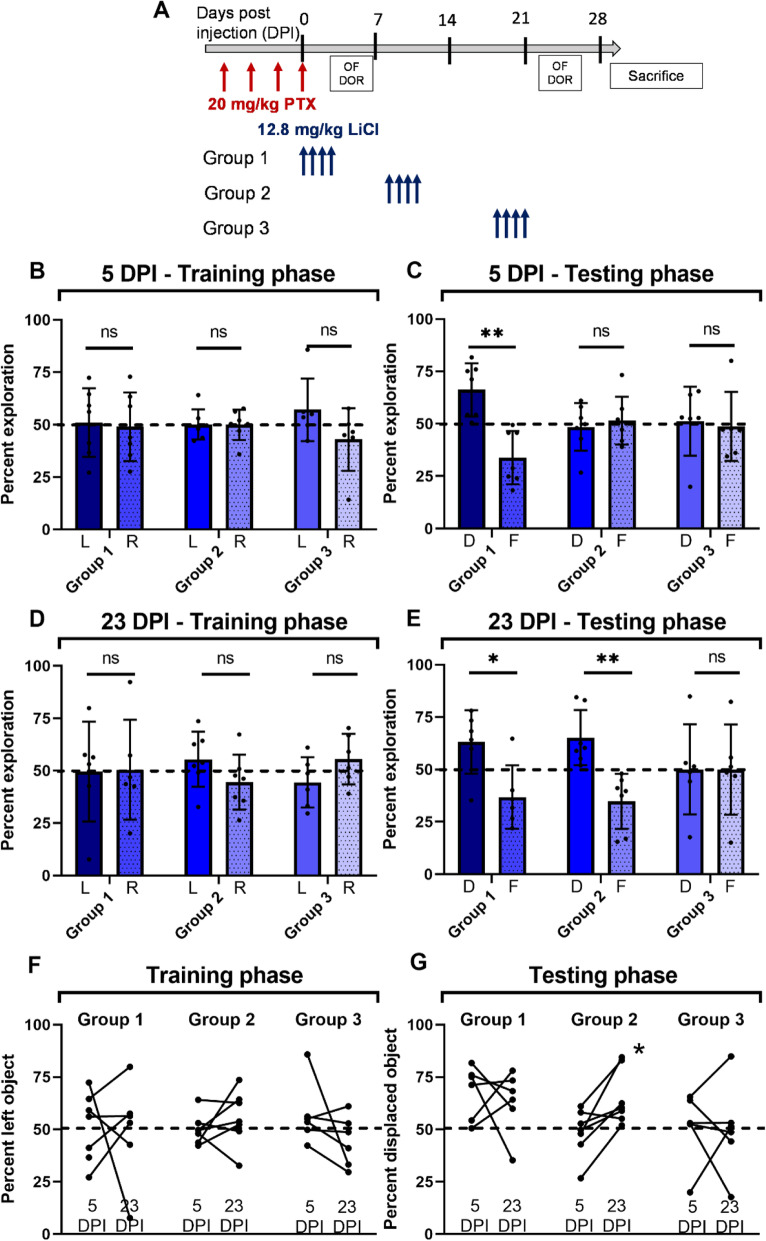
Lithium posttreatment within a limited window reverses paclitaxel-induced short-term memory impairment. (A) Schematic illustration of paclitaxel and lithium injection scheme, followed by behavioral tasks (OFE = open-field exploration, DOR = displaced object recognition, D = displaced, F = familiar). (B-E) Lithium posttreatment within 10 days of paclitaxel injection rescued short-term memory impairment (t-test, adjusted for multiple comparisons). 5 DPI: (B) p > 0.3 for all groups, (C) *p* = 0.001 for group 1, *p* = 0.86 for group 2, p = 0.86 for group 3. 23 DPI: (D) p > 0.3 for all groups, (E) *p* = 0.025 for group 1, p = 0.003 for group 2, *p* = 0.99 for group 3. (F-G): paired t-test. (G) *p* = 0.015 for group 2, *p* > 0.05 for all other groups. *N* = 6–7 mice per group

To investigate whether lithium can also alleviate chemobrain when administered after patients finished chemotherapy and, if yes, what the therapeutic window would be, we investigated several posttreatment schedules in our model. Mice were divided into 3 groups, with all groups receiving 4 × 20 mg/kg paclitaxel. Subsequently, groups received 4 doses of 12.8 mg/kg LiCl at 0–3 DPI, 7–10 DPI, or 17–20 DPI, respectively, and assessed with the established schedule of the DOR task (Fig. 2A). Group 1, which received lithium immediately after paclitaxel, showed normal memory acquisition both at 5 DPI and 23 DPI (Fig. 2B-E). Importantly, group 2, which showed impaired memory acquisition at 5 DPI (Fig. 2C), then received lithium at 7–10 DPI subsequently showed normal memory acquisition when tested at 23 DPI (Fig. 2E). When examined individually, the majority of mice in group 2 developed a significantly greater preference for the displaced object after the lithium treatment (Fig. 2G). However, group 3, which showed impaired memory acquisition at 5 DPI and received lithium more than two weeks after injection, showed no improvement at 23 DPI (Fig. 2G), suggesting that lithium given at this later time point was insufficient to reverse cognitive impairment. As expected, no trend was observed in any group during the training phase (Fig. 2F). These data indicate that, within a limited time window, lithium can not only prevent but also reverse paclitaxel-induced chemobrain.

### Paclitaxel reduces hippocampal neuron complexity

**Fig. 3:**
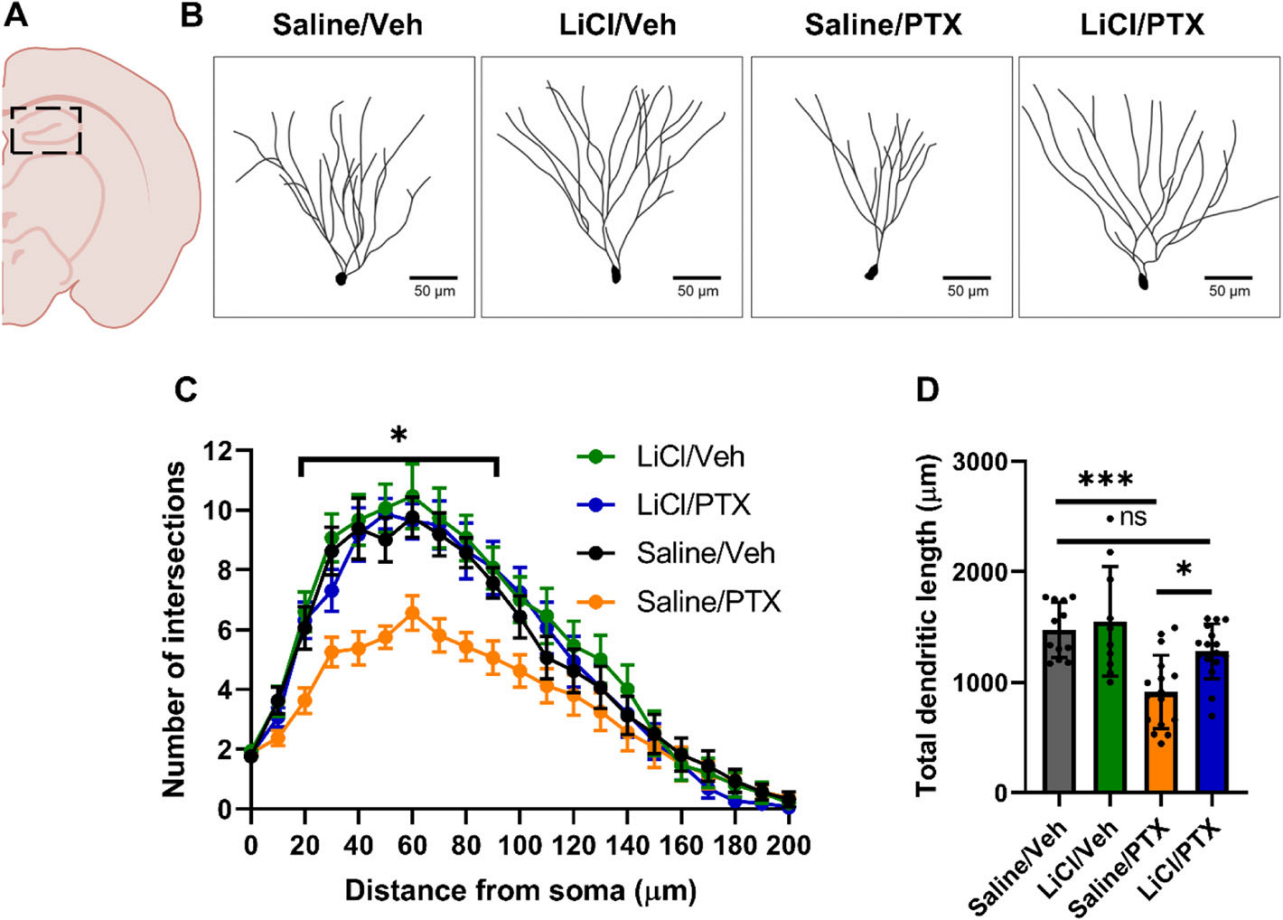
Lithium pretreatment rescues paclitaxel-induced deficits in granule cell morphology. (A) Diagram showing the region where the cells were imaged. (B) Representative traces of granule cells from each group, scale bar shown is 50 μm. (C-D). Paclitaxel treatment reduced granule cell complexity, which could be rescued by lithium pretreatment (C) Repeated measures two-way ANOVA, Distance: F(3.582, 211.4) = 121.0, *p* < 0.0001, Treatment: F [3, 59]=7.653, *p* = 0.0002, Distance x Treatment: F(60, 1180) =2.154, *p* < 0.0001. Dunnet’s multiple comparisons test between Saline/Veh and Saline/PTX: *p* < 0.05 between 20 to 90 μm from the soma. (D) One-way ANOVA: p < 0.0001, Tukey posthoc test: *p* = 0.0003 for Saline/Veh vs. Saline/PTX, p = 0.015 for Saline/PTX vs. LiCl/PTX, *p* = 0.42 for Saline/Veh vs. LiCl/PTX. *N* = 3–4 neurons per mouse, 4–6 mice per group

Next, we investigated possible neuro-morphological correlates underlying chemobrain. Various chemotherapeutics, including cisplatin [51], 5-fluorouracil [52], doxorubicin, and cyclophosphamide [53, 54], were reported to reduce the dendritic complexity in granule cells and CA1 and CA3 pyramidal neurons in the hippocampus. Therefore, we performed Golgi-Cox staining of mouse brain hemispheres, and subsequently, Sholl analysis on dentate gyrus granule cells to examine changes in dendritic complexity as a function of the number of intersections at various radial distances from the cell soma (Fig. 3A-B). Two-way repeated-measures ANOVA revealed a significant effect of treatment, distance, and the interaction distance x treatment on dendritic complexity in the dentate gyrus (Fig. 3C). Post-hoc analysis revealed that saline/paclitaxel neurons showed reduced dendritic arborization compared to saline/vehicle controls, particularly between 20 and 90 μm. In addition, there was a significant reduction in the total dendritic length in the saline/paclitaxel group compared to the other groups (Fig. 3D). No significant differences were found comparing saline/vehicle neurons with lithium/vehicle or lithium/paclitaxel neurons. These results suggest that paclitaxel injections reduced hippocampal dendritic complexity, which was rescued with lithium pretreatment.

### Paclitaxel reduces apical cortical neuron complexity

**Fig. 4:**
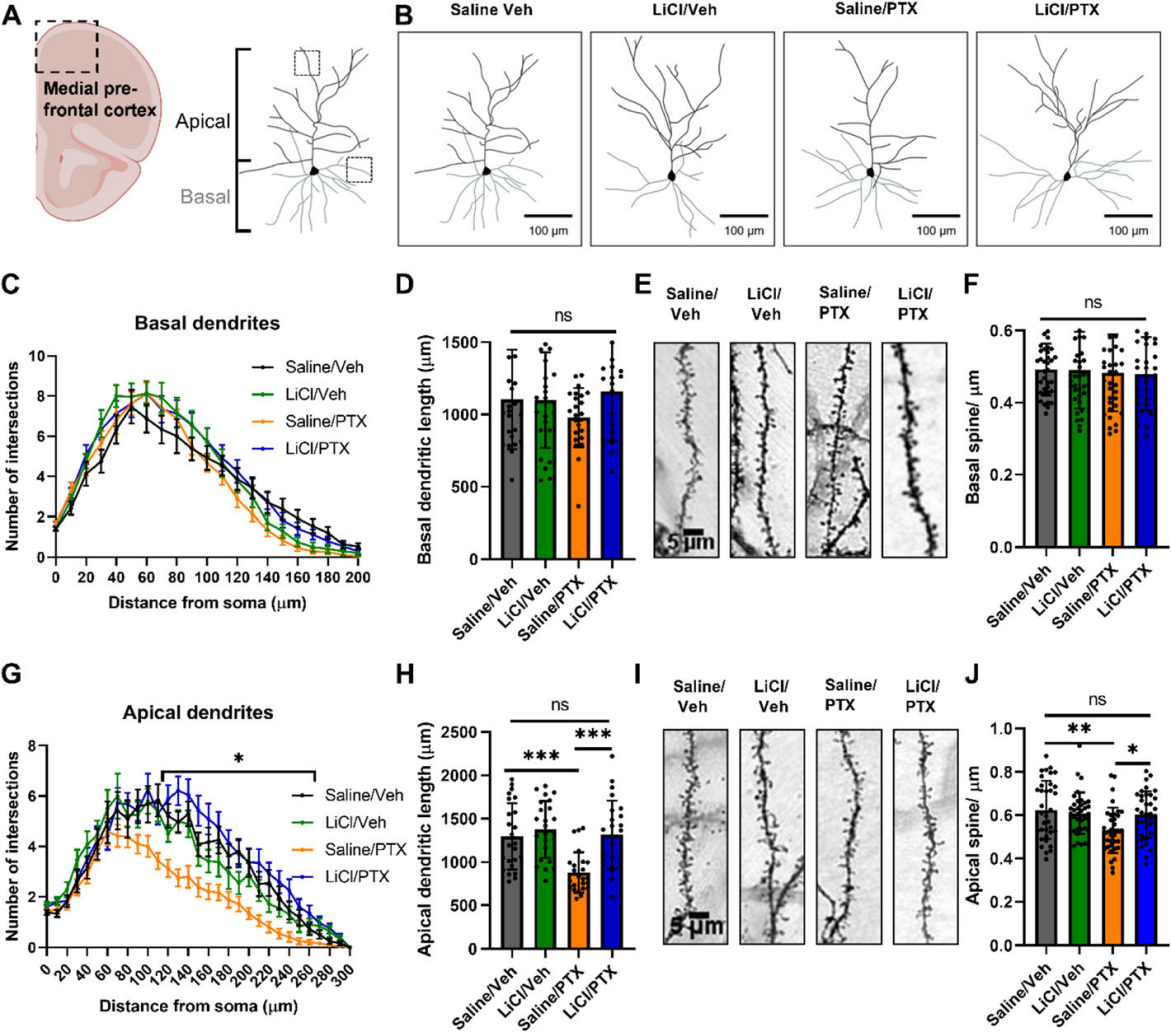
Lithium pretreatment rescues paclitaxel-induced deficits in cortical neuron morphology. (A) Schematic diagram showing the region in the coronal section where cortical neurons were imaged. The dotted boxes indicate the general locations within each neuron where spines were imaged. (B) Representative traces of cortical neurons, basal dendrites are colored grey, apical dendrites are colored black, scale bar shown is 100 μm. (C-J) Paclitaxel treatment reduced the complexity and spine density of apical dendrites, which could be rescued by lithium pretreatment. Basal dendrites and spines: (C) Repeated measures two-way ANOVA, Distance: F(3.555, 327.0) = 148.5, p < 0.0001; Treatment: F(3, 92) = 1.097, *p* = 0.35; Distance x Treatment: F(60, 1840) = 1.286, *p* = 0.071. (D) One-way ANOVA, *p* = 0.23. (E) Representative images of basal spines, scale bar shown is 5 μm. (F) One-way ANOVA, *p* = 0.94. Apical dendrites and spines: (G) Distance: F(4.105, 377.7) = 76.74, p < 0.0001; Treatment: F(3, 92) = 12.32, p < 0.0001; Distance x Treatment: F(90, 2760) = 1.722, p < 0.0001. Dunnet’s multiple comparisons test between Saline/Veh and Saline/PTX: p < 0.05 between 110 to 250 μm from the soma. (H) One-way ANOVA: p < 0.0001, Tukey post-hoc test: p = 0.0003 for Saline/Veh vs. Saline/PTX, *p* = 0.0001 for Saline/PTX vs. LiCl/PTX, p = 0.99 for Saline/Veh vs. LiCl/PTX. (I) Representative images of apical spines, scale bar shown is 5 μm. (J) One-way ANOVA: *p* = 0.0021, Tukey post-hoc test: *p* = 0.0024 for Saline/Veh vs. Saline/PTX, *p* = 0.03 for Saline/PTX vs. LiCl/PTX, *p* = 0.84 for Saline/Veh vs. LiCl/PTX. For Sholl analysis and dendritic lengths, *N* = 4 neurons per mouse, 6 mice per group. For spine density, *n* = 6 segments per mouse, 6 mice per group

As worse performance in our DOR task could suggest impairments in both the frontal cortex and the hippocampus [41], we also performed Golgi-Cox staining and Sholl analysis on layers 2/3 cortical pyramidal neurons in the medial prefrontal cortex (Fig. 4A-B). Because cortical pyramidal neurons exhibit both basal and apical dendrites, each with distinct functions and input sources [55, 56], we performed analyses separately for each region. For basal dendrites, we observed no significant differences in the Sholl analysis and dendritic length among the 4 groups (Fig. 4C-D). Similarly, there were no significant differences in basal spine density (Fig. 4E-F).

In contrast, for apical dendrites, significant differences were observed in the Sholl analysis (Fig. 4G). Post-hoc analysis revealed that saline/paclitaxel neurons showed reduced dendritic arborization compared to saline/vehicle controls, particularly between 110 and 250 μm. Similarly, the saline/paclitaxel group exhibited a significant reduction in apical dendritic length and spine density compared to the other three groups (Fig. H-J). These results suggest that apical dendrites were more susceptible to paclitaxel, whereas basal dendrites were largely spared. Similar results were also observed for neurons in the parietal cortex (Supp. Fig. 5), suggesting that other cortical areas were also affected.

### Paclitaxel upregulates PKCα

**Fig. 5:**
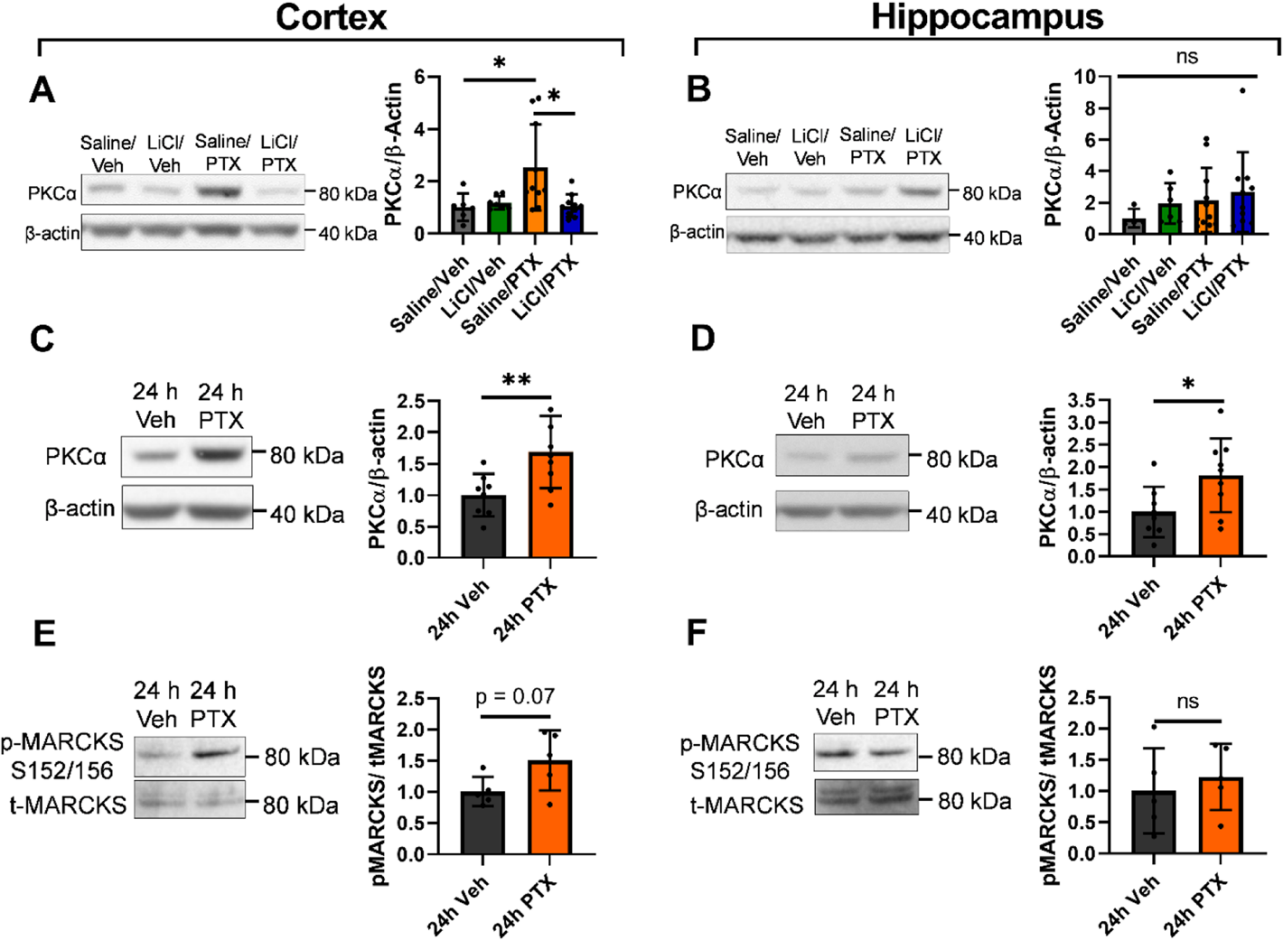
PKCα expression and activity increase primarily in the cortex. (A-B) At 30 DPI, PKCα was upregulated in the cortex, but not in the hippocampus, of the Saline/PTX group. (A) One-way ANOVA, *p* = 0.57. (B) *p* = 0.0059, Tukey post-hoc test: *p* = 0.026 for Saline/Veh vs. Saline/PTX, *p* = 0.042 for Saline/PTX vs. LiCl/PTX, p = 0.99 for Saline/Veh vs. LiCl/PTX. (C-F) PKCα was upregulated both in the hippocampus and cortex after a single paclitaxel injection. (C) Two-tailed t-test, p = 0.03, (D) *p* = 0.0096, (E) *p* = 0.58, (F) *p* = 0.068. N = 4–10 mice per group for chronic expression. N = 4–8 per group for acute expression

Next, we focused on molecular changes that may underly chemobrain. Particularly, we investigated changes in the InsP3R pathway, which we hypothesized to be dysregulated by paclitaxel [20–22, 26]. We observed an upregulation in PKCα, an effector of the InsP3R pathway, in the cortex of mice treated with paclitaxel at 30 DPI (Fig. 5A), but not in the hippocampus (Fig. 5B). No changes were observed for other proteins involved in the InsP3R pathway, including InsP3R1, NCS1, and phospholipase C (PLC-β1) (Supp. Fig. 6).

To assess molecular changes involved in the initiation of chemobrain, we also collected tissues from mice 24 h after a single injection of 20 mg/kg paclitaxel injection or vehicle control. An upregulation in PKCα was again observed in both the cortex and the hippocampus (Fig. 5C-D). To measure possible downstream functional consequences of PKCα activity, we examined the phosphorylated form of the PKC substrate myristoylated alanine-rich C-kinase substrate (MARCKS) [57]. There was a trend towards increased pMARCKS (S152/156) in the cortex 24 h after paclitaxel injection (Fig. 5E), but not in the hippocampus (Fig. 5F). Taken together, our molecular analyses suggest that PKCα contributes to the behavioral and cellular deficits in mice receiving paclitaxel.

### Pretreatment with PKC inhibitor chelerythrine rescues paclitaxel-induced impairment in short-term memory and neuronal morphology

**Fig. 6:**
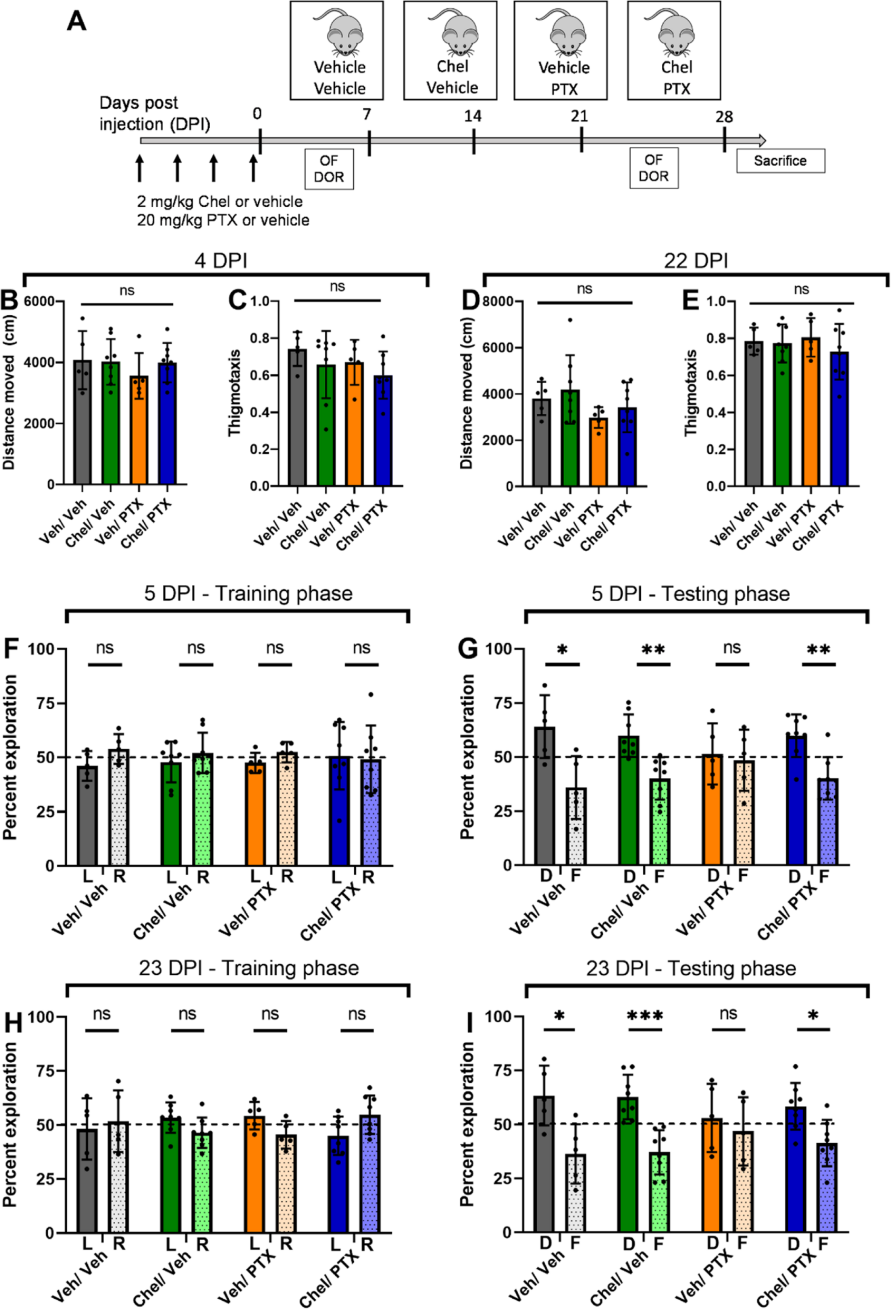
PKC inhibitor chelerythrine pretreatment rescues paclitaxel-induced short-term memory impairment. (A) Schematic illustration of paclitaxel and chelerythrine injection, followed by behavioral tasks (OF = open-field exploration, DOR = displaced object recognition, D = displaced, F = familiar, Chel = chelerythrine). (B-E) Locomotor activity and anxiety were similar across all groups at 4 and 22 DPI. One-way ANOVA, p > 0.3 for all comparisons. (F-I) Chelerythrine pretreatment rescued paclitaxel-induced cognitive impairment. (F-I): t-test, adjusted for multiple comparisons. 5 DPI: (F) p > 0.3 for all groups, (G) *p* = 0.031 for Veh/Veh, *p* = 0.0047 for Chel/Veh, *p* = 0.75 for Veh/PTX, p = 0.0047 for Chel/PTX. 23 DPI: (H) *p* > 0.15 for all groups. (I) *p* = 0.029 for Veh/Veh, *p* = 0.0008 for Chel/Veh, *p* = 0.55 for Veh/PTX, *p* = 0.022 for Chel/PTX. *N* = 5–8 mice per group

It was previously shown that chronic restraint stress in rats resulted in calcium-dependent activation of PKC activity, leading to reduced cortical spines and dendrites, and hence impaired working memory [58]. Furthermore, in the same study, pretreatment with a brain-penetrant PKC inhibitor, chelerythrine, rescued the impaired working memory [58]. Therefore, to test the hypothesis that increased PKC expression and activity contribute to paclitaxel-induced memory impairment, we examined whether pretreatment with chelerythrine could prevent memory deficits in our model of chemobrain. Pretreatment with chelerythrine resulted in similar results to pretreatment with lithium (Fig. 6). First, no differences in total distance moved and thigmotaxis were observed among the four groups at both 4 DPI and 22 DPI (Fig. 6A-E). Second, pretreatment with chelerythrine prevented paclitaxel-induced memory impairment at both 5 DPI and 23 DPI, whereas chelerythrine alone did not affect memory acquisition (Fig. 6F-I).

**Fig. 7:**
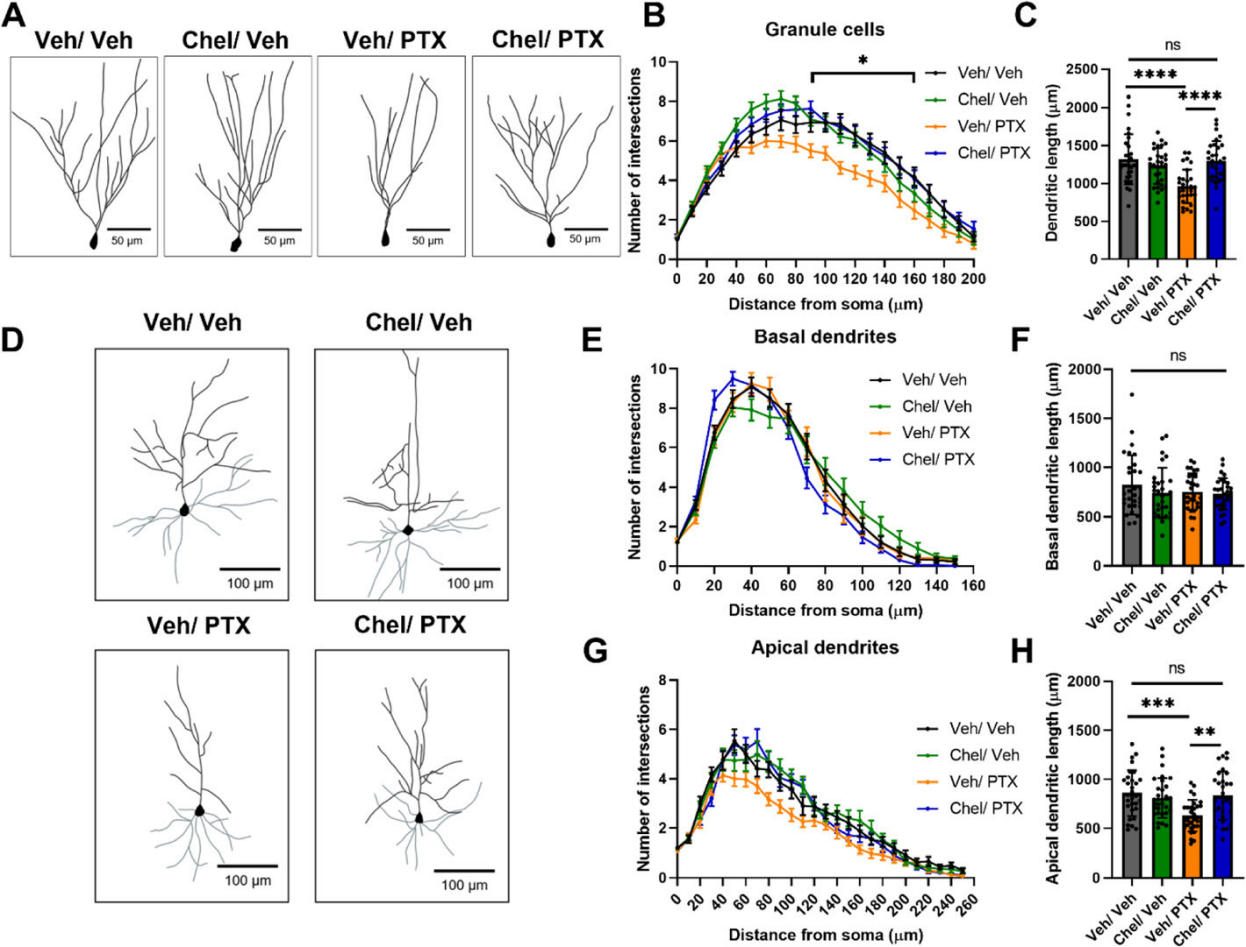
PKC inhibitor chelerythrine pretreatment rescues paclitaxel-induced deficits in neuronal morphology. (A) Representative traces of granule cells in the dentate gyrus, scale bar shown is 50 μm. (B-C) Paclitaxel treatment reduced the complexity and length of granule cells of the dentate gyrus, which could be rescued by chelerythrine pretreatment. (B) Repeated measures two-way ANOVA, Distance: F(3.451, 400.3) = 174.6, p < 0.0001; Treatment: F(3, 116) = 5.600, *p* = 0.0013; Distance x Treatment: F(60, 2320) = 2.413, p < 0.0001. Dunnet’s multiple comparisons test between Chel/Veh and Chel/PTX: p < 0.05 between 90 and 160 μm from the soma. (C) One-way ANOVA: followed by Tukey post-hoc test: p < 0.0001 for Veh/Veh vs. Veh/PTX, p = 0.001 for Veh/PTX vs. Chel/PTX, *p* = 0.98 for Veh/Veh vs. Chel/PTX. (D) Representative traces of cortical neurons, basal dendrites are colored grey, apical dendrites are colored black, scale bar shown is 100 μm. (E-G) Paclitaxel treatment reduced the complexity and length of apical dendrites of layers 2/3 cortical pyramidal neurons in the medial prefrontal cortex, which could be rescued by chelerythrine pretreatment. (E) Distance: F(3.961, 459.5) = 457.9, p < 0.0001; Treatment: F(3, 116) = 0.9192, *p* = 0.43; Distance x Treatment: F(45, 1740) = 1.366, *p* = 0.055. (F) One-way ANOVA, *p* = 0.41. (G) Distance: F(6.408, 743.4) = 152.9, p < 0.0001; Treatment: F(3, 116) = 5.895, *p* = 0.0009; Distance x Treatment: F(75, 2900) = 1.587, *p* = 0.0011. p < 0.05 at 50, 80, and 160 μm from the soma. (F) p = 0.0003 for Veh/Veh vs. Veh/PTX, *p* = 0.007 for Veh/PTX vs. Chel/PTX, *p* = 0.97 for Veh/Veh vs. Chel/PTX. *N* = 6 neurons per mouse, 5 mice per group

We further investigated the effects of chelerythrine pretreatment and paclitaxel on hippocampal and neuronal morphology. Similar to previously observed (Figs. 3 and 4), paclitaxel treatment reduced the complexity and dendritic length of granule cells in the hippocampus and apical dendrites of neurons in the cortex, whereas basal dendrites were spared (Fig. 7A-H). These results further underscore that chelerythrine and lithium act in a similar pathway to rescue paclitaxel-induced short-term memory impairment.

## Discussion

### Lithium for the prevention and treatment of paclitaxel-induced cognitive impairment

Here, we successfully established that treatment with lithium both before and after paclitaxel injections rescued cognitive impairment. Our results agree with previous studies reporting that lithium pretreatment rescues paclitaxel-induced peripheral neuropathy [26, 59] and cognitive impairment [32]. To the best of our knowledge, we are the first to report that posttreatment with lithium, albeit within a limited time window, can reverse the cognitive deficits induced by paclitaxel. We speculate that the window of effectiveness of lithium treatment matches the trajectories of the mechanisms underlying paclitaxel-induced cognitive deficits. Similar to traumatic brain injury, chemobrain is initiated by an acute insult, which is the administration of paclitaxel [60]. This initiating phase is then followed by the chronic phase, in which deficits are consolidated and maintained even when the original insult is gone. The mechanisms of action of lithium remain varied and incompletely understood. Although we previously showed that lithium blocks paclitaxel-induced InsP3R calcium oscillation [21], lithium also inhibits inositol monophosphatase [29] and PKC [61, 62] to further downregulate the InsP3R calcium pathway. Lithium appears to interfere with the initiation and consolidation of chemobrain, and is less effective over time as the cellular and molecular deficits become permanent. An alternative explanation is that in our experimental design (Fig. 2A), group 3 received lithium only 3 days before the chronic DOR task, but more time would be needed between lithium treatment and DOR task before improvements can be observed. Further experiments will be needed to clarify this question.

### A role for PKC hyperactivation in cellular and behavioral deficits

We observed a reduction in dendritic complexity and length in the hippocampus and cortex of mice treated with paclitaxel, which we hypothesize to be the cellular mechanism for the memory deficits observed in these mice. The upregulation of PKCα acutely in the hippocampus and the cortex, which persists chronically in the cortex, may provide the underlying molecular mechanism for this observation. PKCα activity can also be activated by elevation in calcium [63]. PKCα hyperactivity has been implicated in stress and age-induced loss of dendritic and spinal complexity and cognitive deficits [58, 63, 64]. Furthermore, PKC isoforms have been shown to play a role in paclitaxel-induced peripheral neuropathy [65]. We also observed the trend towards the upregulation of a PKC substrate, pMARCKS. Phosphorylation of MARCKS has been shown to cause dendrite and spine loss through inducing actin instability [66]. Interestingly, paclitaxel, but not other chemotherapeutic drugs, was shown to dose-dependently increase pMARCKS in breast cancer cell lines [67]. As a proof of concept, we showed that pretreatment with the PKC inhibitor chelerythrine also rescued deficits in neuronal morphology and memory acquisition in animals receiving paclitaxel.

### Region-specific vulnerability to chemotherapy

We previously proposed that determining the specific cognitive modalities, anatomical regions, and cell populations that are more vulnerable to chemotherapy will be essential for discovering prevention and treatment options [60]. Interestingly, although paclitaxel was reported to preferentially accumulate in the hippocampus compared to the cortex [32], we found that neuronal morphology was also altered in the cortex. In the prefrontal cortex, apical dendrites and spine density were reduced, whereas basal dendrites and spines were spared. Persistent activity in layer 2/3 apical dendrites was proposed to be essential for recurrent neuronal activity, which in turn sustains working memory and attention [68, 69] – cognitive functions that are often impaired in chemobrain. A similar phenomenon of apical vulnerability has been frequently reported in animal models of stress-induced cognitive impairment [58, 70–73], as well as AD [74] and aging [75]. Apical dendrites receive input from diverse sources such as higher cortical regions and the thalamus, and function to modulate selectivity [56]. In contrast, basal dendrites receive input from more local sources such as local pyramidal cells and interneurons, and function to drive stimulus preference [56]. Although the cause of selective apical vulnerability remains to be clarified, candidates include differential distribution of molecular machineries, for example, availability of channels and receptors, and altered input into basal or apical dendrites. Our findings suggest that loss of apical spines and dendrites is a neural correlate for chemobrain, and may share similar pathways with cognitive deficits in aging, AD, and psychological stress. In addition to intracellular selective vulnerabilities, some cell populations are likely to be more vulnerable to chemotherapeutic agents than others. For example, CA1/3 hippocampal neurons were also shown to be damaged by the chemotherapeutic 5-fluorouracil [52]. We observed paclitaxel-induced neuromorphological impairment in the frontal cortex, parietal cortex, and dentate gyrus. Further determination of region- and population-specific vulnerability will inform both possible neural correlates for the symptoms of chemobrain and targets for neuroprotective treatment.

### Model, limitations, and future directions

**Fig. 8:**
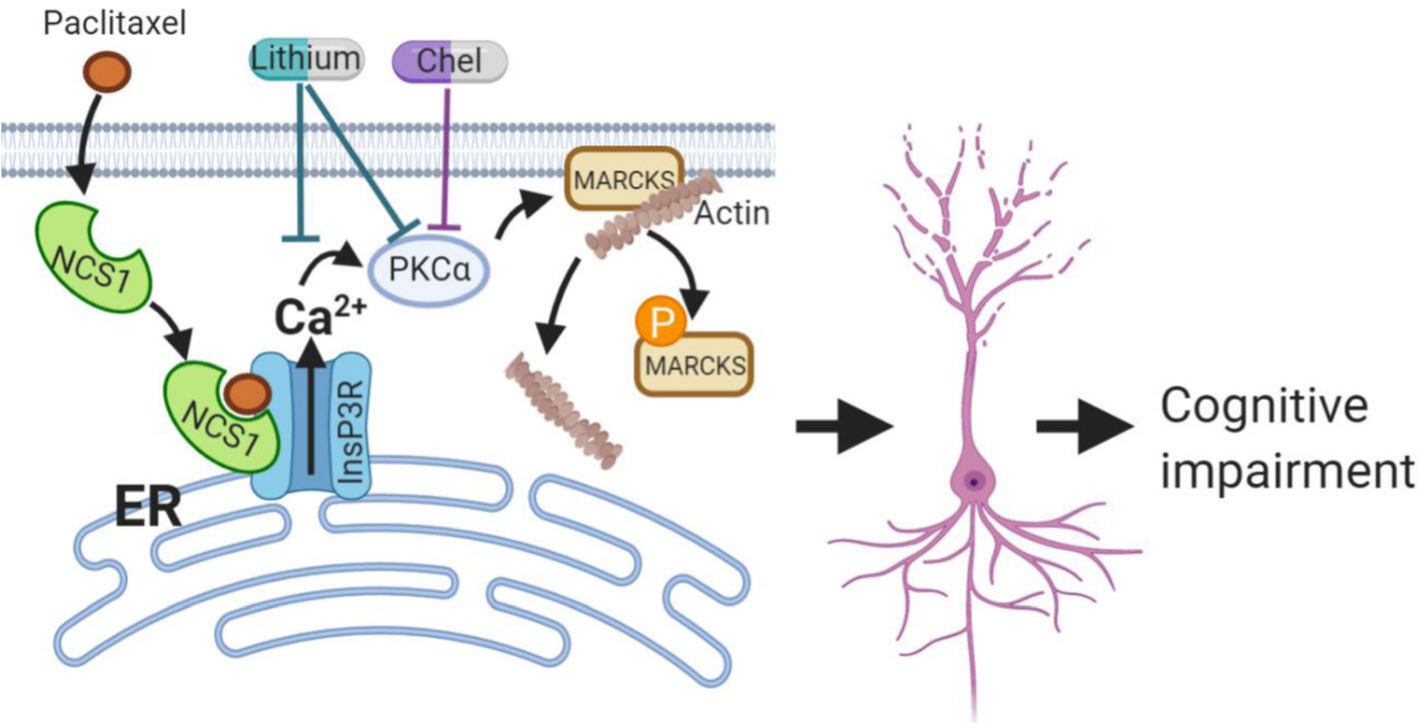
Proposed model for the mechanism of paclitaxel-induced cognitive impairment. Paclitaxel binding to NCS1 enhanced NCS1 binding to the InsP3R resulting in increased calcium release from the ER into the cytoplasm. The increase in calcium concentration, as well as an upregulation of PKCα, leads to PKC hyperactivity. PKCα, in turn, phosphorylates MARCKS into pMARCKS, leading to actin instability. This instability subsequently leads to loss of spines and dendrites, and hence cognitive impairment. Lithium, through inhibiting InsP3R-dependent calcium release and PKCα, and chelerythrine (Chel), through inhibiting PKCα, can rescue paclitaxel-induced cognitive impairment

Combining behavioral, cellular, and molecular observations, we propose a model for paclitaxel-induced cognitive impairment and describe how lithium and chelerythrine can interfere with this pathway (Fig. 8). First, paclitaxel binding to NCS1 leads to increased calcium oscillation from the InsP3R [20, 21, 24, 27]. This results in the upregulation and activation of PKC, which phosphorylates MARCKS into pMARCKS, leading to actin instability, and hence spine and dendrite retraction. Lithium interferes with this pathway by depleting InsP3 to decrease InsP3R activity, or altering InsP3R/NCS1 interaction, or indirectly blocking PKC activity. Chelerythrine blocks PKC activation and hence blocks MARCKS phosphorylation. Interfering in this pathological pathway rescued dendrite and spine retraction, and consequently prevented memory impairment.

A limitation of our study is that we utilized young, healthy female mice that received paclitaxel as IP injections. However, cancer patients receive paclitaxel as IV injections or infusions and often have comorbidities that increase their risks of developing chemobrain, including advanced age, tumor burden, and psychological stress. Therefore, for future studies, we propose utilizing animal models that better reflect chemobrain, such as aged or tumor-bearing mice receiving IV paclitaxel infusion that mimics standard treatment regimens [60]. Both male and female animals should also be used to determine if there are sex differences in developing chemobrain and potential neuroprotective rescue by lithium. Additionally, we observed the neuroprotective effect of lithium at the lowest dose tested of 12.8 mg/kg. As lithium can be toxic at high doses, leading to renal failure, tremors, and altered consciousness [76], identifying the minimum effective lithium dose will help minimize these risks.

Collectively, our findings suggest a pathway for paclitaxel-induced cognitive impairment. Paclitaxel, and potentially other chemotherapeutic drugs, may accelerate neurodegeneration through InsP3R-dependent calcium release, a common pathway for cognitive impairment in aging, psychological stress, and Alzheimer’s disease [33, 34]. Although the mechanisms of lithium-based therapy remain incompletely understood, its pharmacokinetics are well-studied, and low lithium may be beneficial and feasible for chemobrain as it has been shown to be generally neuroprotective [29]. Taken together, lithium and PKC inhibitors may be good preventions and treatments for chemobrain in cancer survivors.

## Supplementary Information

**Additional file 1 Supp.** Fig. 1 **Optimization of paclitaxel injection and lithium pretreatment.** (A) Schematic illustration for paclitaxel and lithium injection, followed by behavioral tasks (OF = open-field exploration, DOR = displaced object recognition, *n* = 5 mice per group). (B) Weight was measured daily before and after paclitaxel injection and normalized to the first day of injection. The red triangles indicated days with paclitaxel injection. Mice lost approximately 5–10% of their body weights after 2 injections but quickly recovered afterward. (C-D) At 5 and 23 DPI, paclitaxel-only mice did not discriminate between the objects (*p* = 0.54 and *p* = 0.48 respectively). Mice receiving both paclitaxel and 4 × 12.8 mg/kg LiCl spent significantly more time exploring the displaced object compared to the familiar object on both days (both *p* < 0.005). Mice receiving both paclitaxel and 8 × 12.8 mg/kg LiCl or 4 × 25.6 mg/kg LiCl showed mixed results. *N* = 5 mice per group

**Additional file 2 Supp.** Fig. 2 **Efficacious dose of lithium is below the common therapeutic range.** Mouse plasma lithium level following a 12.8 mg/kg intraperitoneal injection of lithium. Plasma lithium peaked at 0.36 mM, which is below the lower therapeutic target range (0.5 to 0.8 mM) in humans. Lithium is almost cleared out from the system 6 h after injection. *N* = 3–4 mice for each time point. Blood samples were obtained through cardiac puncture. Lithium concentration was measure by Yale Laboratory Medicine using a colorimetric assay

**Additional file 3 Supp.** Fig. 3 **Weights are not different among the 4 groups**. Weight was measured daily before and after paclitaxel injection and normalized to the first day of injection. The red triangles indicated days with paclitaxel injection. Mice lost approximately 5% of their body weights during injections but quickly recovered afterwards. No significant differences among groups were found (mixed ANOVA with correction of repeated measures, group factor = 0.08). *N* = 7-17 mice per group

**Additional file 4 Supp.** Fig. 4 **Total interaction time is not affected by treatments.** (A-H) One-way ANOVA, *p* > 0.05 for all plots

**Additional file 5 Supp.** Fig. 5 **Golgi-Cox staining and quantification of layers 2/3 cortical pyramidal neurons in the parietal cortex 30 DPI.** (A) Schematic diagram showing the region in the coronal section where cortical neurons were imaged, and (B) their representative images. (C-F) Analysis showed that there were no differences in basal dendritic complexity (repeated measures two-way ANOVA), dendritic length, or spine density (one-way ANOVA) among the four groups. (G) Sholl analysis revealed a substantial reduction in apical dendritic complexity in the group receiving saline and paclitaxel (repeated measures two-way ANOVA). Lithium pretreatment rescued the reduction to the level comparable to those of the two groups receiving vehicle control. (H-J) Similarly, compared to other groups, apical dendrites from the group treated with saline and paclitaxel showed a significant reduction in dendritic length and spine density (one-way ANOVA, followed by Tukey post-hoc test). *N* = 3 to 4 neurons each from 4 to 6 mice per group for Sholl analysis and dendritic length. For spine density, *N*= 6 segments per mouse, 4–6 mice per group

**Additional file 6 Supp.** Fig. 6 **Expression levels of various proteins in the InsP3R pathway are unchanged 30 DPI.** (A) Representative blots from hippocampus samples and quantification. (B) Representative blot from cortex samples and quantification. For all graphs, *p* > 0.05 (one-way ANOVA). N = 3–10 mice per group, with each dot representing a sample from a mouse. For the representative image, note that not all proteins shown were from the same preparation. However, each protein was normalized to the β-actin lane from the same preparation

**Additional file 7 Supp. Table 1: List of primary antibodies used**

**Additional file 8 Supp. Table 2: Detailed statistical analyses for** Fig. 1-7

**Additional file 9 Original files for traced images**

## Data Availability

The data and materials are available from the corresponding author upon request.

## Abbreviations

AD: Alzheimer’s disease
CNS: central nervous system
DOR: displaced object recognition
DPI: days post injection
InsP3R: inositol 1,4,5-trisphosphate receptor
MARCKS: myristoylated alanine-rich C-kinase substrate
NCS1: neuronal calcium sensor 1
OFE: open-field exploration
PKC: protein kinase C
PTX: paclitaxel
