# Supplementary material for: Pharmacological rescue of cognitive function in a mouse model of chemobrain
Source: Mol Neurodegener. 2021 Jun 26;16:41. doi: 10.1186/s13024-021-00463-2 (PMC8235868; doi:10.1186/s13024-021-00463-2)
Supplement: Supplementary file 1 — Additional file 1 Supp. Fig. 1 Optimization of paclitaxel injection and lithium pretreatment. (A) Schematic illustration for paclitaxel and lithium injection, followed by behavioral tasks (OF = open-field exploration, DOR = displaced object recognition, n = 5 mice per group). (B) Weight was measured daily before and after paclitaxel injection and normalized to the first day of injection. The red triangles indicated days with paclitaxel injection. Mice lost approximately 5–10% of their body weights after 2 injections but quickly recovered afterward. (C-D) At 5 and 23 DPI, paclitaxel-only mice did not discriminate between the objects (p = 0.54 and p = 0.48 respectively). Mice receiving both paclitaxel and 4 × 12.8 mg/kg LiCl spent significantly more time exploring the displaced object compared to the familiar object on both days (both p < 0.005). Mice receiving both paclitaxel and 8 × 12.8 mg/kg LiCl or 4 × 25.6 mg/kg LiCl showed mixed results. N = 5 mice per group [file 13024_2021_463_MOESM1_ESM.docx]

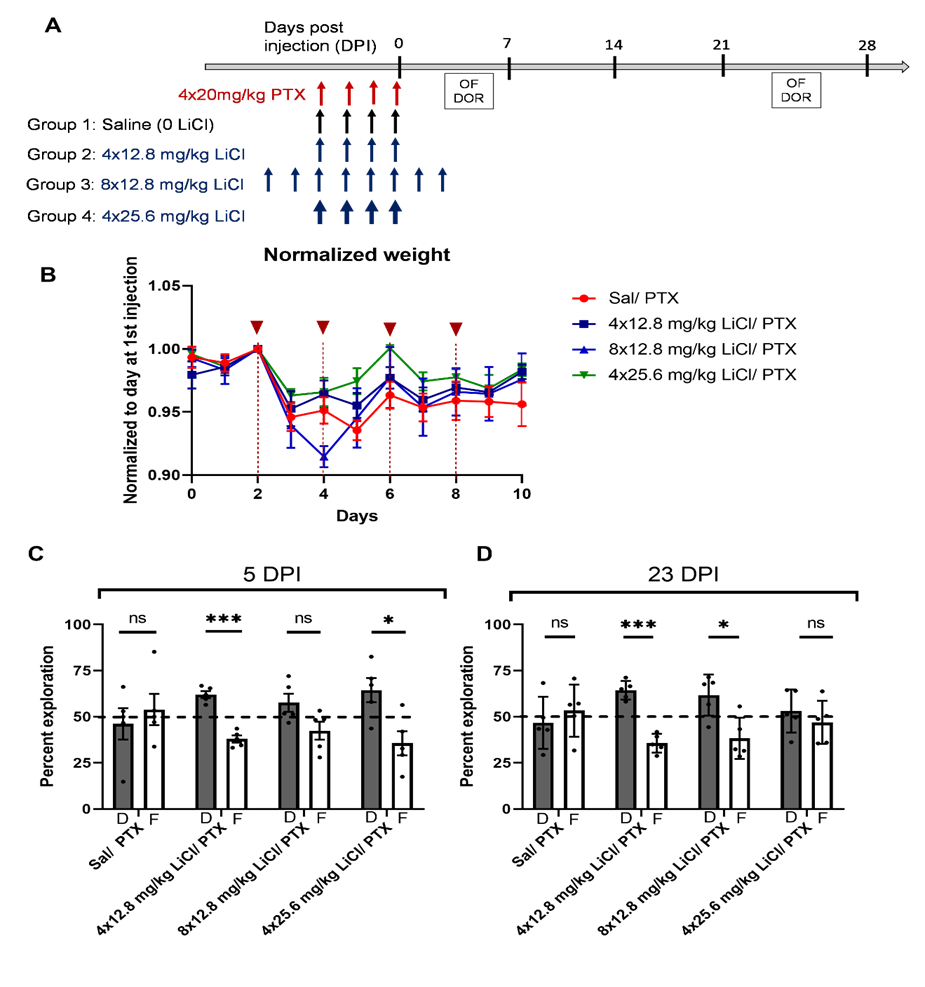


**Supp. Fig. 1 Optimization of paclitaxel injection and lithium pretreatment.** (A) Schematic illustration for paclitaxel and lithium injection, followed by behavioral tasks (OF = open-field exploration, DOR = displaced object recognition, n = 5 mice per group). (B) Weight was measured daily before and after paclitaxel injection and normalized to the first day of injection. The red triangles indicated days with paclitaxel injection. Mice lost approximately 5-10% of their body weights after 2 injections but quickly recovered afterward. (C-D) At 5 and 23 DPI, paclitaxel-only mice did not discriminate between the objects (p = 0.54 and p = 0.48 respectively). Mice receiving both paclitaxel and 4 x 12.8 mg/kg LiCl spent significantly more time exploring the displaced object compared to the familiar object on both days (both p < 0.005). Mice receiving both paclitaxel and 8 x 12.8 mg/kg LiCl or 4 x 25.6 mg/kg LiCl showed mixed results. N = 5 mice per group.
