# Supplementary material for: Pharmacological rescue of cognitive function in a mouse model of chemobrain
Source: Mol Neurodegener. 2021 Jun 26;16:41. doi: 10.1186/s13024-021-00463-2 (PMC8235868; doi:10.1186/s13024-021-00463-2)
Supplement: Supplementary file 2 — Additional file 2 Supp. Fig. 2 Efficacious dose of lithium is below the common therapeutic range. Mouse plasma lithium level following a 12.8 mg/kg intraperitoneal injection of lithium. Plasma lithium peaked at 0.36 mM, which is below the lower therapeutic target range (0.5 to 0.8 mM) in humans. Lithium is almost cleared out from the system 6 h after injection. N = 3–4 mice for each time point. Blood samples were obtained through cardiac puncture. Lithium concentration was measure by Yale Laboratory Medicine using a colorimetric assay [file 13024_2021_463_MOESM2_ESM.docx]

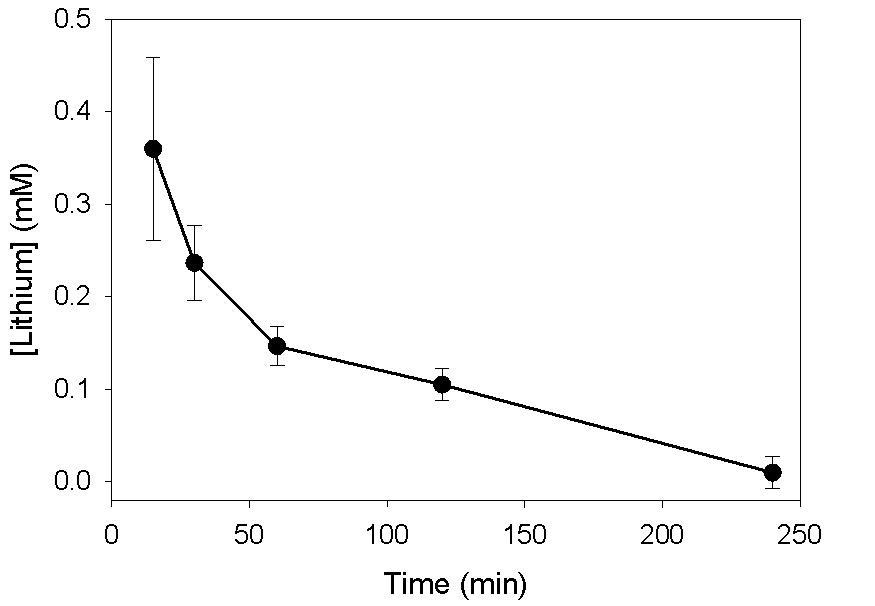


**Supp. Fig. 2 Efficacious dose of lithium is below the common therapeutic range.** Mouse plasma lithium level following a 12.8mg/kg intraperitoneal injection of lithium. Plasma lithium peaked at 0.36 mM, which is below the lower therapeutic target range (0.5 to 0.8 mM) in humans. Lithium is almost cleared out from the system 6 hours after injection. N = 3-4 mice for each time point. Blood samples were obtained through cardiac puncture. Lithium concentration was measure by Yale Laboratory Medicine using a colorimetric assay.
