# Supplementary material for: Pharmacological rescue of cognitive function in a mouse model of chemobrain
Source: Mol Neurodegener. 2021 Jun 26;16:41. doi: 10.1186/s13024-021-00463-2 (PMC8235868; doi:10.1186/s13024-021-00463-2)
Supplement: Supplementary file 3 — Additional file 3 Supp. Fig. 3 Weights are not different among the 4 groups. Weight was measured daily before and after paclitaxel injection and normalized to the first day of injection. The red triangles indicated days with paclitaxel injection. Mice lost approximately 5% of their body weights during injections but quickly recovered afterwards. No significant differences among groups were found (mixed ANOVA with correction of repeated measures, group factor = 0.08). N = 7-17 mice per group [file 13024_2021_463_MOESM3_ESM.docx]

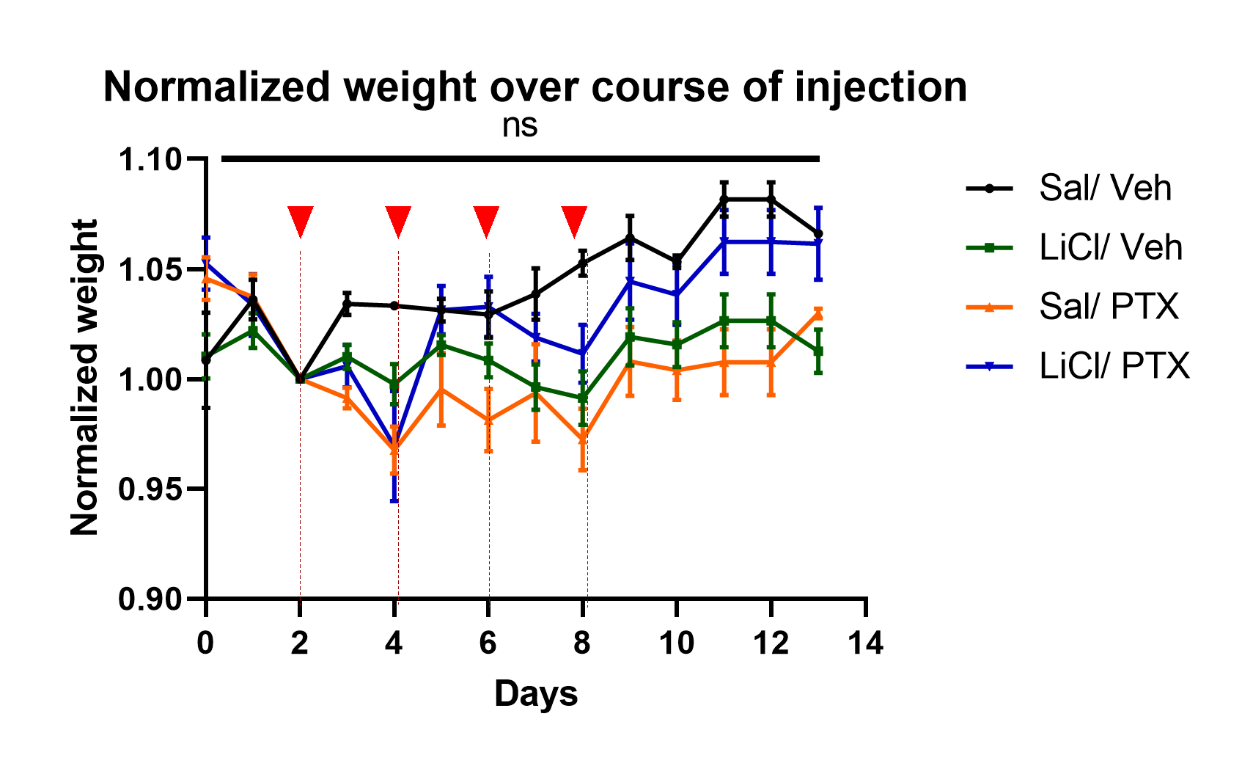


**Supp. Fig. 3 Weights are not different among the 4 groups**. Weight was measured daily before and after paclitaxel injection and normalized to the first day of injection. The red triangles indicated days with paclitaxel injection. Mice lost approximately 5% of their body weights during injections but quickly recovered afterward. No significant differences among groups were found (mixed ANOVA with correction of repeated measures, group factor = 0.08). N =717 mice per group.
