# Supplementary figures and images for: Pharmacological rescue of cognitive function in a mouse model of chemobrain
Source: Mol Neurodegener. 2021 Jun 26;16:41. doi: 10.1186/s13024-021-00463-2 (PMC8235868; doi:10.1186/s13024-021-00463-2)

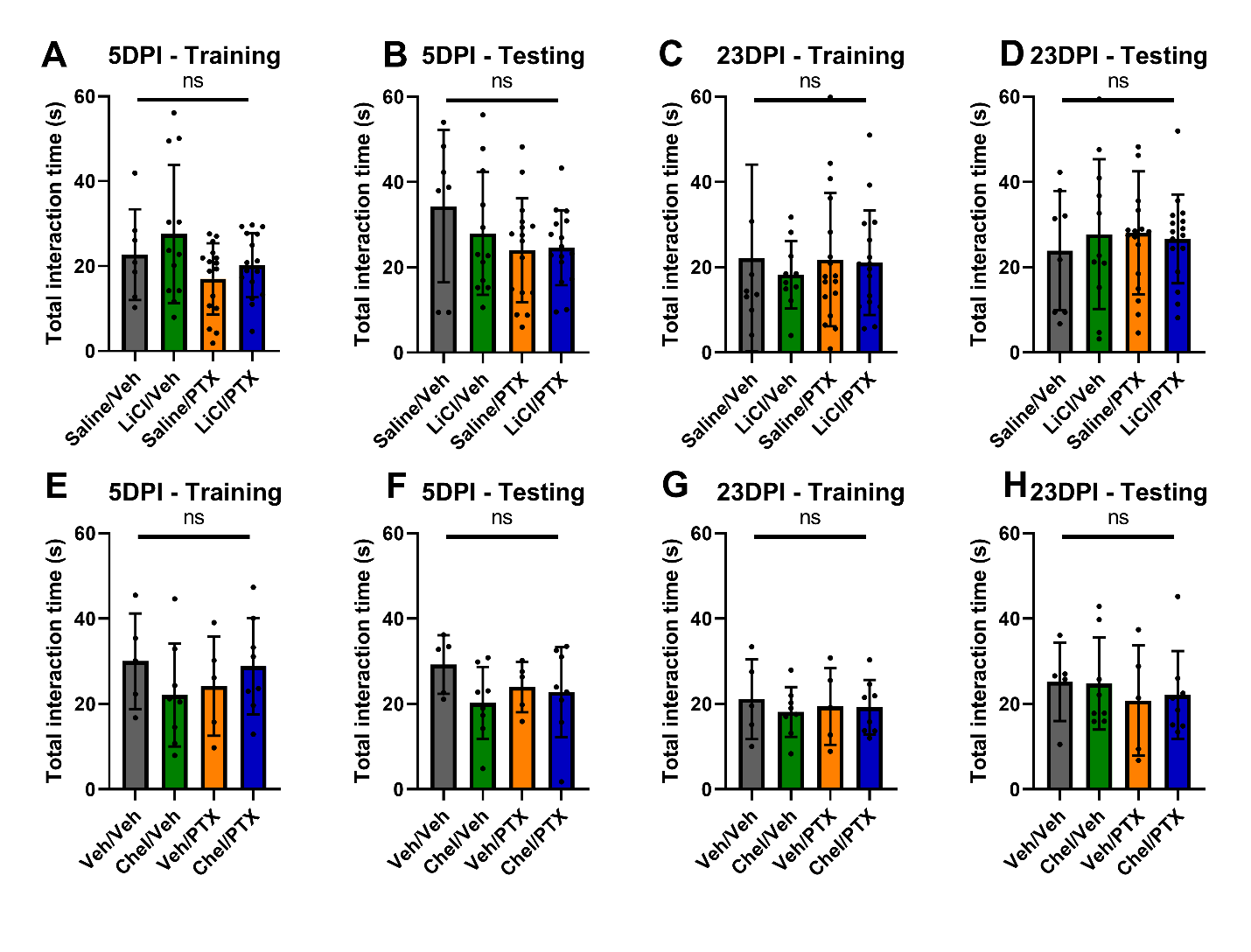


**Supp. Fig. 4 Total interaction time is not affected by treatments.** (A-H) One-way ANOVA, p > 0.0.5 for all plots.

Supplement: Supplementary file 4 — Additional file 4 Supp. Fig. 4 Total interaction time is not affected by treatments. (A-H) One-way ANOVA, p > 0.05 for all plots [file 13024_2021_463_MOESM4_ESM.docx]
