# Supplementary material for: Pharmacological rescue of cognitive function in a mouse model of chemobrain
Source: Mol Neurodegener. 2021 Jun 26;16:41. doi: 10.1186/s13024-021-00463-2 (PMC8235868; doi:10.1186/s13024-021-00463-2)
Supplement: Supplementary file 5 — Additional file 5 Supp. Fig. 5 Golgi-Cox staining and quantification of layers 2/3 cortical pyramidal neurons in the parietal cortex 30 DPI. (A) Schematic diagram showing the region in the coronal section where cortical neurons were imaged, and (B) their representative images. (C-F) Analysis showed that there were no differences in basal dendritic complexity (repeated measures two-way ANOVA), dendritic length, or spine density (one-way ANOVA) among the four groups. (G) Sholl analysis revealed a substantial reduction in apical dendritic complexity in the group receiving saline and paclitaxel (repeated measures two-way ANOVA). Lithium pretreatment rescued the reduction to the level comparable to those of the two groups receiving vehicle control. (H-J) Similarly, compared to other groups, apical dendrites from the group treated with saline and paclitaxel showed a significant reduction in dendritic length and spine density (one-way ANOVA, followed by Tukey post-hoc test). N = 3 to 4 neurons each from 4 to 6 mice per group for Sholl analysis and dendritic length. For spine density, N= 6 segments per mouse, 4–6 mice per group [file 13024_2021_463_MOESM5_ESM.docx]

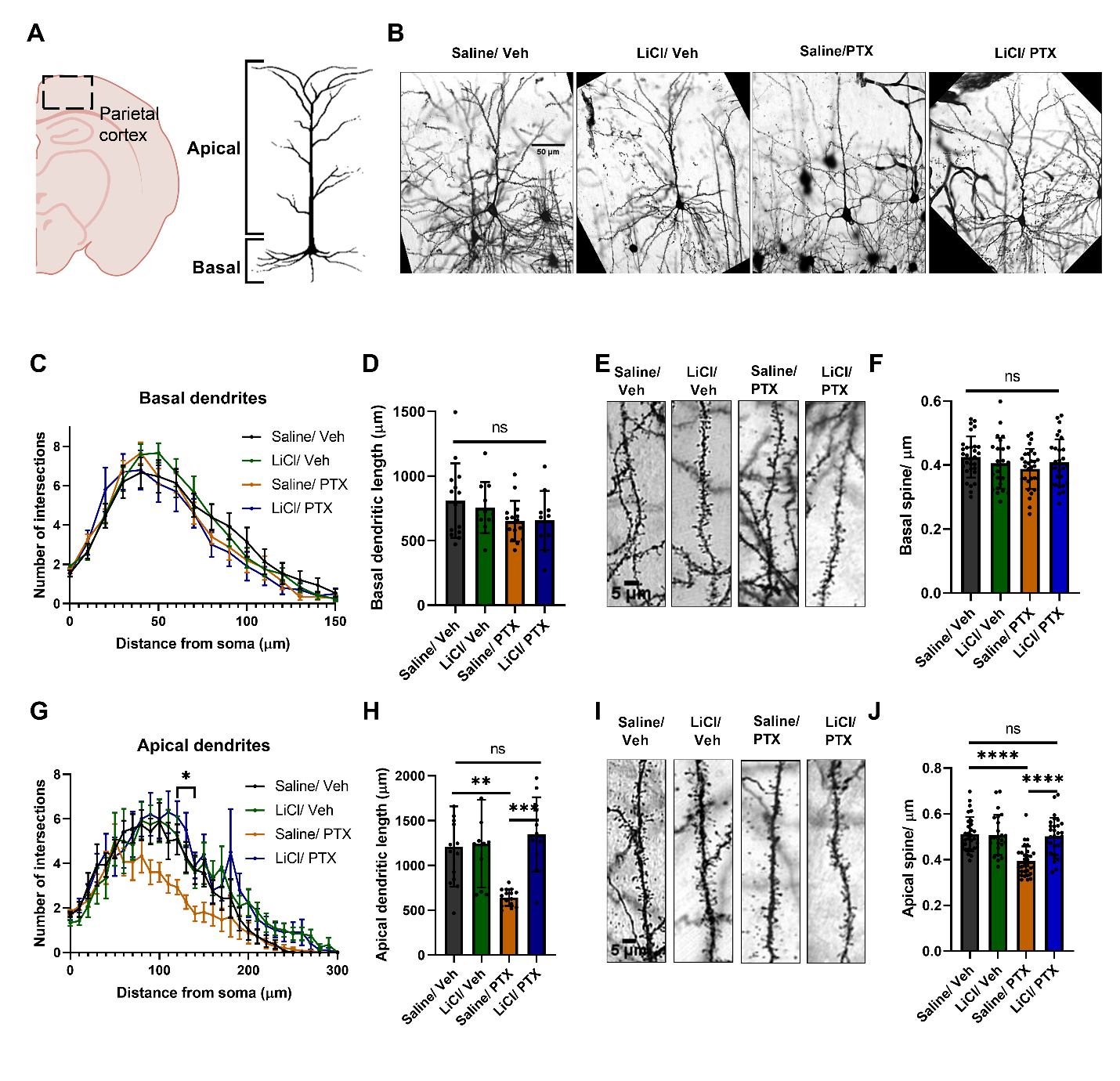


**Supp. Fig. 5 Golgi-Cox staining and quantification of layers 2/3 cortical pyramidal neurons in the parietal cortex 30 DPI.** (A) Schematic diagram showing the region in the coronal section where cortical neurons were imaged, and (B) their representative images. (C-F) Analysis showed that there were no differences in basal dendritic complexity (repeated measures two-way ANOVA), dendritic length, or spine density (one-way ANOVA) among the four groups. (G) Sholl analysis revealed a substantial reduction in apical dendritic complexity in the group receiving saline and paclitaxel (repeated measures two-way ANOVA). Lithium pretreatment rescued the reduction to the level comparable to those of the two groups receiving vehicle control. (H-J) Similarly, compared to other groups, apical dendrites from the group treated with saline and paclitaxel showed a significant reduction in dendritic length and spine density (one-way ANOVA, followed by Tukey post-hoc test). N = 3 to 4 neurons each from 4-6 mice per group for Sholl analysis and dendritic length. For spine density, n = 6 segments per mouse, 4-6 mice per group.
