# Supplementary material for: Pharmacological rescue of cognitive function in a mouse model of chemobrain
Source: Mol Neurodegener. 2021 Jun 26;16:41. doi: 10.1186/s13024-021-00463-2 (PMC8235868; doi:10.1186/s13024-021-00463-2)
Supplement: Supplementary file 6 — Additional file 6 Supp. Fig. 6 Expression levels of various proteins in the InsP3R pathway are unchanged 30 DPI. (A) Representative blots from hippocampus samples and quantification. (B) Representative blot from cortex samples and quantification. For all graphs, p > 0.05 (one-way ANOVA). N = 3–10 mice per group, with each dot representing a sample from a mouse. For the representative image, note that not all proteins shown were from the same preparation. However, each protein was normalized to the β-actin lane from the same preparation [file 13024_2021_463_MOESM6_ESM.docx]

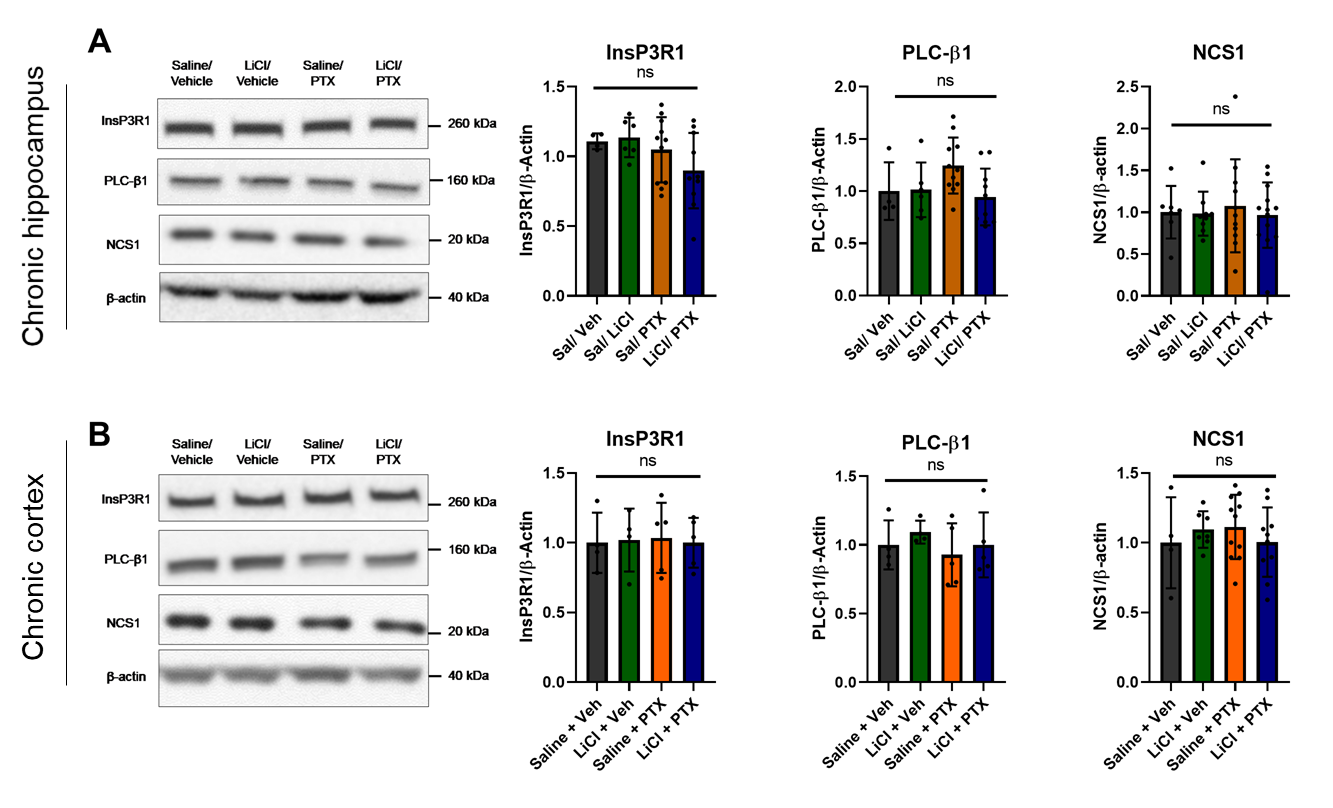


**Supp. Fig. 6 Expression levels of various proteins in the InsP3R1 pathway are unchanged 30 DPI.** (A) Representative blots from hippocampus samples and quantification. (B) Representative blot from cortex samples and quantification. For all graphs, p > 0.05 (one-way ANOVA). N = 3-10 mice per group, with each dot representing a sample from a mouse. For the representative image, note that not all proteins shown were from the same preparation. However, each protein was normalized to the β-actin lane from the same preparation.
