# Supplementary material for: Pharmacological rescue of cognitive function in a mouse model of chemobrain
Source: Mol Neurodegener. 2021 Jun 26;16:41. doi: 10.1186/s13024-021-00463-2 (PMC8235868; doi:10.1186/s13024-021-00463-2)
Supplement: Supplementary file 7 — Additional file 7 Supp. Table 1: List of primary antibodies used [file 13024_2021_463_MOESM7_ESM.docx]

**Supp. Table 1: List of primary antibodies used**

| **Antibody** | **Source** | **Identifier** |
| --- | --- | --- |
| β-Actin | Cell Signaling | 8H10D10 |
| NCS1 | Santa Cruz | FL-190 |
| InsP3R1 | Home-made | YU272 |
| PKCα | Sigma Aldrich | P5704 |
| pMARCKS (Ser152/156) | Cell Signaling | 2741S |
| MARCKS | Cell Signaling | 5607S |
| PLC-β1 | Santa Cruz | sc-5291 |
