# Supplementary material for: Pharmacological rescue of cognitive function in a mouse model of chemobrain
Source: Mol Neurodegener. 2021 Jun 26;16:41. doi: 10.1186/s13024-021-00463-2 (PMC8235868; doi:10.1186/s13024-021-00463-2)
Supplement: Supplementary file 8 — Additional file 8 Supp. Table 2: Detailed statistical analyses for Fig. 1-7 [file 13024_2021_463_MOESM8_ESM.docx]

**Supp. Table 2: Detailed statistical analyses for Fig. 1-7**

| **Fig.** | **Statistical test** | **p-value** | **Adjusted p-value/**  **Post-hoc pairwise comparison** |
| --- | --- | --- | --- |
| **FIG. 1** | | | |
| 1B | One-way ANOVA | F=0.82, p=0.49, R^2^=0.056 |  |
| 1C | One-way ANOVA | F=0.31, p=0.82, R^2^=0.021 |  |
| 1D | One-way ANOVA | F=0.42, p=0.74, R^2^=0.03 |  |
| 1E | One-way ANOVA | F=0.99, p=0.41, R^2^=0.07 |  |
| 1F | Unpaired t-test, corrected for multiple comparisons |  | Saline/Veh: p=0.68  LiCl/Veh: p=0.60  Saline/PTX: p=0.68  LiCl/PTX: p=0.22 |
| 1G | Unpaired t-test, corrected for multiple comparisons |  | Saline/Veh: p=0.0044  LiCl/Veh: p = 0.0026  Saline/PTX: p=0.41  LiCl/PTX: p=0.0026 |
| 1H | Unpaired t-test, corrected for multiple comparisons |  | Saline/Veh: p=0.69  LiCl/Veh: p=0.75  Saline/PTX: p=0.49  LiCl/PTX: p=0.32 |
| 1G | Unpaired t-test, corrected for multiple comparisons |  | Saline/Veh: p=0.047  LiCl/Veh: p=0.005  Saline/PTX: p=0.79  LiCl/PTX: p<0.0001 |
| **FIG. 2** | | | |
| 2B | Unpaired t-test, corrected for multiple comparisons |  | Group 1: p=0.97  Group 2: p=0.98  Group 3: p=0.34 |
| 2C | Unpaired t-test, corrected for multiple comparisons |  | Group 1: p=0.0014  Group 2: p=0.86  Group 3: p=0.86 |
| 2D | Unpaired t-test, corrected for multiple comparisons |  | Group 1: p=0.95  Group 2: p=0.36  Group 3: p=0.36 |
| 2E | Unpaired t-test, corrected for multiple comparisons |  | Group 1: p=0.025  Group 2: p=0.003  Group 3: p=0.99 |
| 2F | Paired t-test |  | Group 1: p=0.73  Group 2: p=0.46  Group 3: p=0.20 |
| 2F | Paired t-test |  | Group 1: p=0.63  Group 2: p=0.015  Group 3: p=0.90 |
| **FIG. 3** | | | |
| 3C | Two-way repeated-measures ANOVA, followed by Dunnett's multiple comparisons test | Distance: p<0.0001,  F(3.582, 211.4)=121.0, 56.9% variation  Treatment: p=0.0002,  F(3, 59)=7.653, 3.52% variation  Distance x Treatment: p<0.0001, F(60, 1180)=2.154, 3.04% variation | Saline/Veh vs. Saline/PTX: p < 0.05 between 20 and 90 μm. |
| 3D | One-way ANOVA, followed by Tukey's multiple comparisons test | F=10.17, p<0.0001, R^2^ = 0.38 | Saline/Veh vs. LiCl/Veh: p=0.95  Saline/Veh vs. Saline/PTX: p=0.0003  Saline/Veh vs. LiCl/PTX: p=0.42  LiCl/Veh vs. Saline/PTX: p<0.0001  LiCl/Veh vs. LiCl/PTX: p=0.1910  Saline/PTX vs. LiCl/PTX: p=0.015 |
| **FIG. 4** | | | |
| 4C | Two-way repeated-measures ANOVA | Distance: p<0.0001,  F (3.555, 327.0)=148.5, 53.3% variation  Treatment p=0.35,  F(3, 92)=1.097, 0.42% variation  Distance x Treatment:  p=0.071, F(60, 1840)=1.286, 1.4% variation |  |
| 4D | One-way ANOVA | F=1.454, p=0.23, R^2^=0.045 |  |
| 4F | One-way ANOVA | F=0.128, p=0.94, R^2^=0.003 |  |
| 4G | Two-way repeated-measures ANOVA, followed by Dunnett's multiple comparisons test | Distance: p<0.0001,  F(4.105, 377.7)=76.74, 37.9% variation  Treatment: p<0.0001,  F(3, 92)=12.32, 4.07% variation  Distance x Treatment:  p<0.0001, F(90, 2760)=1.722, 2.55% variation | Saline/Veh vs. Saline/PTX: p < 0.05 between 110 and 250 μm |
| 4H | One-way ANOVA, followed by Tukey's multiple comparisons test | F=10.76, p<0.0001, R^2^=0.26 | Saline/Veh vs. LiCl/Veh: p=0.85  Saline/Veh vs. Saline/PTX: p=0.0003  Saline/Veh vs. LiCl/PTX: p=0.99  LiCl/Veh vs. Saline/PTX: p<0.0001  LiCl/Veh vs. LiCl/PTX: p=0.92  Saline/PTX vs. LiCl/PTX: p=0.0001 |
| 4J | One-way ANOVA, followed by Tukey's multiple comparisons test | F=5.156, p = 0.0021, R^2^=0.1 | Saline/Veh vs. LiCl/Veh: p=0.93  Saline/Veh vs. Saline/PTX: p=0.0024  Saline/Veh vs. LiCl/PTX: p=0.84  LiCl/Veh vs. Saline/PTX: p=0.016  LiCl/Veh vs. LiCl/PTX: p=0.99  Saline/PTX vs. LiCl/PTX: p=0.031 |
| **FIG. 5** | | | |
| 5A | One-way ANOVA, followed by Tukey's multiple comparisons test | F=5.101, p=0.0059, R^2^=0.35 | Saline/Veh vs. LiCl/Veh: p=0.98  Saline/Veh vs. Saline/PTX: p=0.0262  Saline/Veh vs. LiCl/PTX: p=0.99  LiCl/Veh vs. Saline/PTX: p=0.0423  LiCl/Veh vs. LiCl/PTX: p=0.99  Saline/PTX vs. LiCl/PTX: p=0.011 |
| 5B | One-way ANOVA | F=0.68, p=0.57, R^2^=0.07 |  |
| 5C | Two-tailed t-test | p=0.0096 |  |
| 5D | Two-tailed t-test | p=0.033 |  |
| 5E | Two-tailed t-test | p=0.07 |  |
| 5F | Two-tailed t-test | p=0.58 |  |
| **FIG. 6** | | | |
| 6B | One-way ANOVA | F=0.52, p=0.67, R^2^=0.066 |  |
| 6C | One-way ANOVA | F=1.04. p=0.39, R^2^=0.12 |  |
| 6D | One-way ANOVA | F=0.14, p=0.26, R^2^=0.16 |  |
| 6E | One-way ANOVA | F=0.53, p=0.69, R^2^=0.067 |  |
| 6F | Unpaired t-test, corrected for multiple comparisons |  | Veh/Veh: p=0.37  Chel/Veh: p=0.62  Veh/PTX: p=0.37  Chel/PTX: p=0.84 |
| 6G | Unpaired t-test, corrected for multiple comparisons |  | Veh/Veh: p=0.031  Chel/Veh: p=0.0047  Veh/PTX: p=0.75  Chel/PTX: p=0.047 |
| 6H | Unpaired t-test, corrected for multiple comparisons |  | Veh/Veh: p=0.71  Chel/Veh: p=0.17  Veh/PTX: p=0.17  Chel/PTX: p=0.17 |
| 6G | Unpaired t-test, corrected for multiple comparisons |  | Veh/Veh: p=0.029  Chel/Veh: p=0.00077  Veh/PTX: p=0.55  Chel/PTX: p=0.026 |
| **FIG. 7** | | | |
| 7B | Two-way repeated-measures ANOVA, followed by Dunnett's multiple comparisons test | Distance: p<0.0001,  F(3.451, 400.3)=174.6, 47.76% variation  Treatment: p=0.0013,  F(3, 116)=5.600, 2.35% variation  Distance x Treatment:  p<0.0001, F(60, 2320)=2.413, 1.98% variation | Veh/Veh vs. Veh/PTX: p<0.05 between 90 and 160 μm. |
| 7C | One-way ANOVA, followed by Tukey's multiple comparisons test | F=11.13, p<0.0001, R^2^=0.22 | Veh/Veh vs. Chel/Veh: p=0.56  Veh/Veh vs. Veh/PTX: p<0.0001  Veh/Veh vs. Chel/PTX: p=0.98  Chel/Veh vs. Veh/PTX: p=0.001  Chel/Veh vs. Chel/PTX: p=0.81  Veh/PTX vs. Chel/PTX: p<0.0001 |
| 7E | Two-way repeated-measures ANOVA | Distance: p<0.0001,  F(3.961, 459.5)=457.9, 71% variation  Treatment: p=0.43,  F(3, 116)=0.9192, 0.24% variation  Distance x Treatment:  P=0.055, F(45, 1740)=1.366, 0.63% variation |  |
| 7F | One-way ANOVA | F=0.97, P=0.41, R^2^=0.024 |  |
| 7G | Two-way repeated-measures ANOVA, followed by Dunnett's multiple comparisons test | Distance: p<0.0001,  F(6.408, 743.4)=152.9, 50.2% variation  Treatment: p=0.0009,  F(3, 116)=5.895, 1.34% variation  Distance x Treatment:  P=0.0011, F (75, 2900) = 1.587, 1.56% variation | Veh/Veh vs. Veh/PTX: p<0.05 at 50, 90 and 160 μm. |
| 7H | One-way ANOVA, followed by Tukey's multiple comparisons test | F=7.19, p=0.0002, R^2^=0.16 | Veh/Veh vs. Chel/Veh: p=0.81  Veh/Veh vs. Veh/PTX: p=0.0003  Veh/Veh vs. Chel/PTX: p=0.97  Chel/Veh vs. Veh/PTX: p=0.0073  Chel/Veh vs. Chel/PTX: p=0.97  Veh/PTX vs. Chel/PTX: p=0.0018 |
