# Supplementary material for: Pharmacological rescue of cognitive function in a mouse model of chemobrain
Source: Mol Neurodegener. 2021 Jun 26;16:41. doi: 10.1186/s13024-021-00463-2 (PMC8235868; doi:10.1186/s13024-021-00463-2)

**Additional materials**

**Original files for traced images**


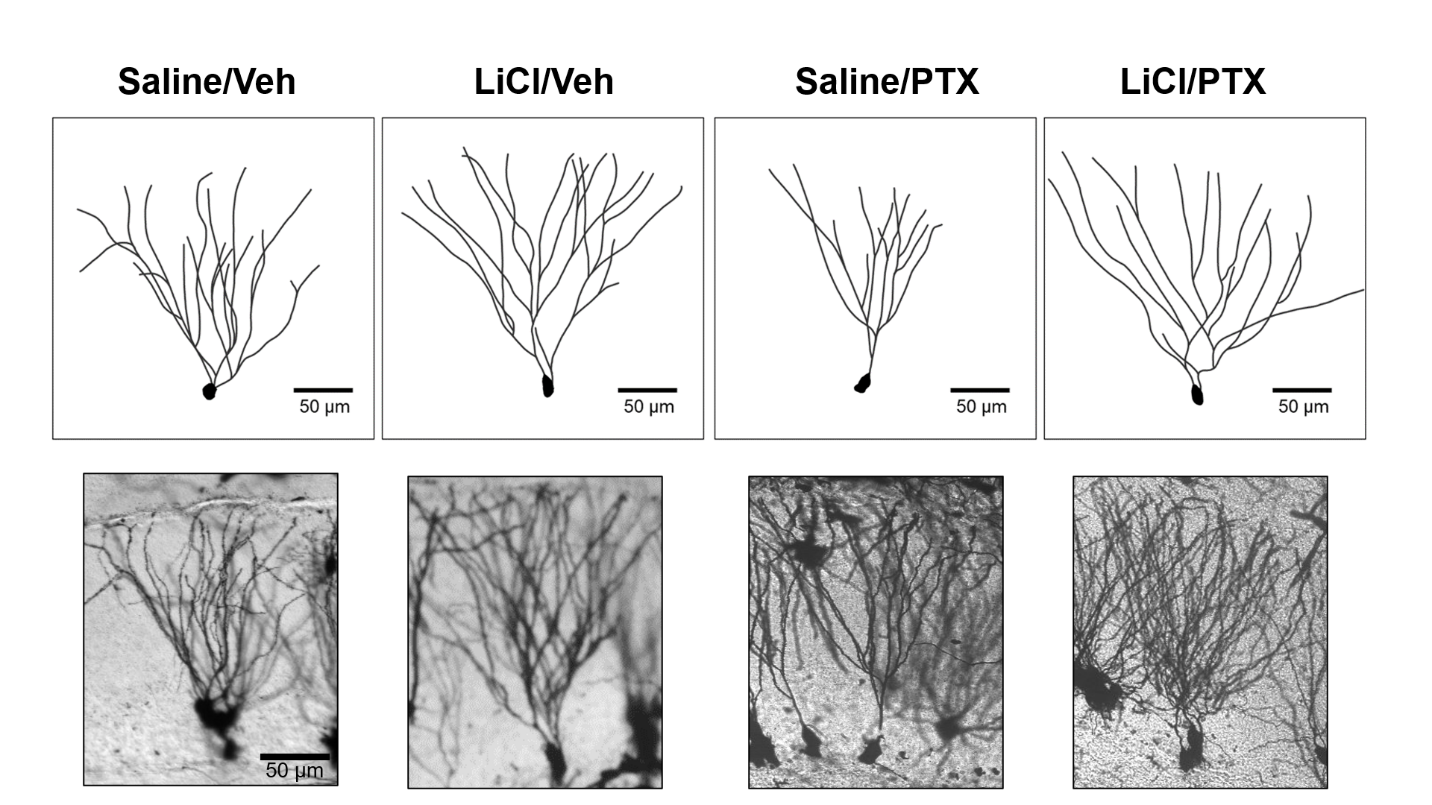
Ref, Fig. 3

Ref. Fig. 4

**
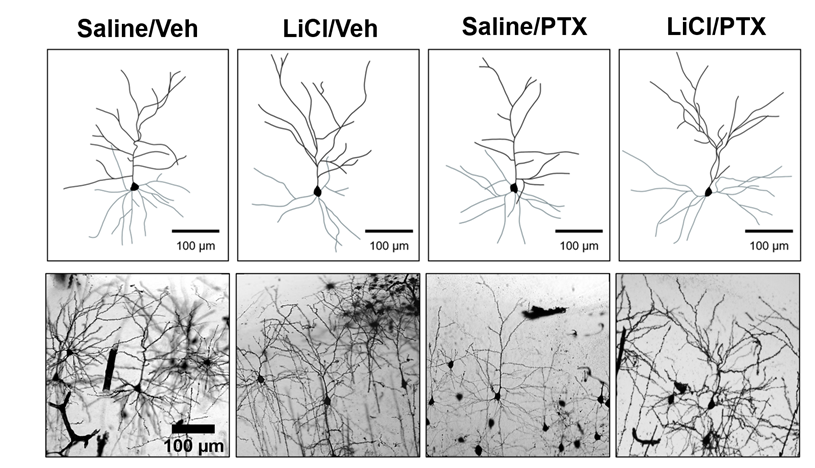
**

Ref. Fig. 7


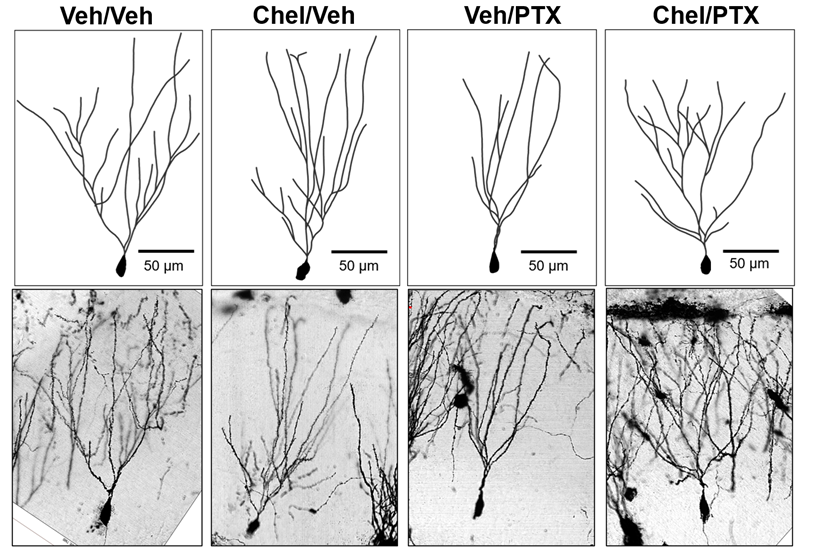


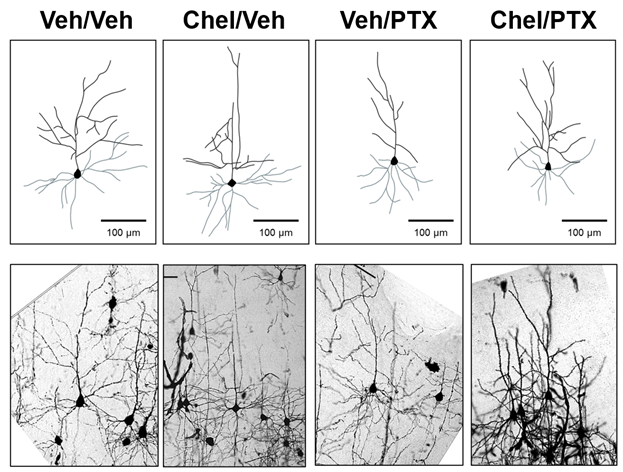


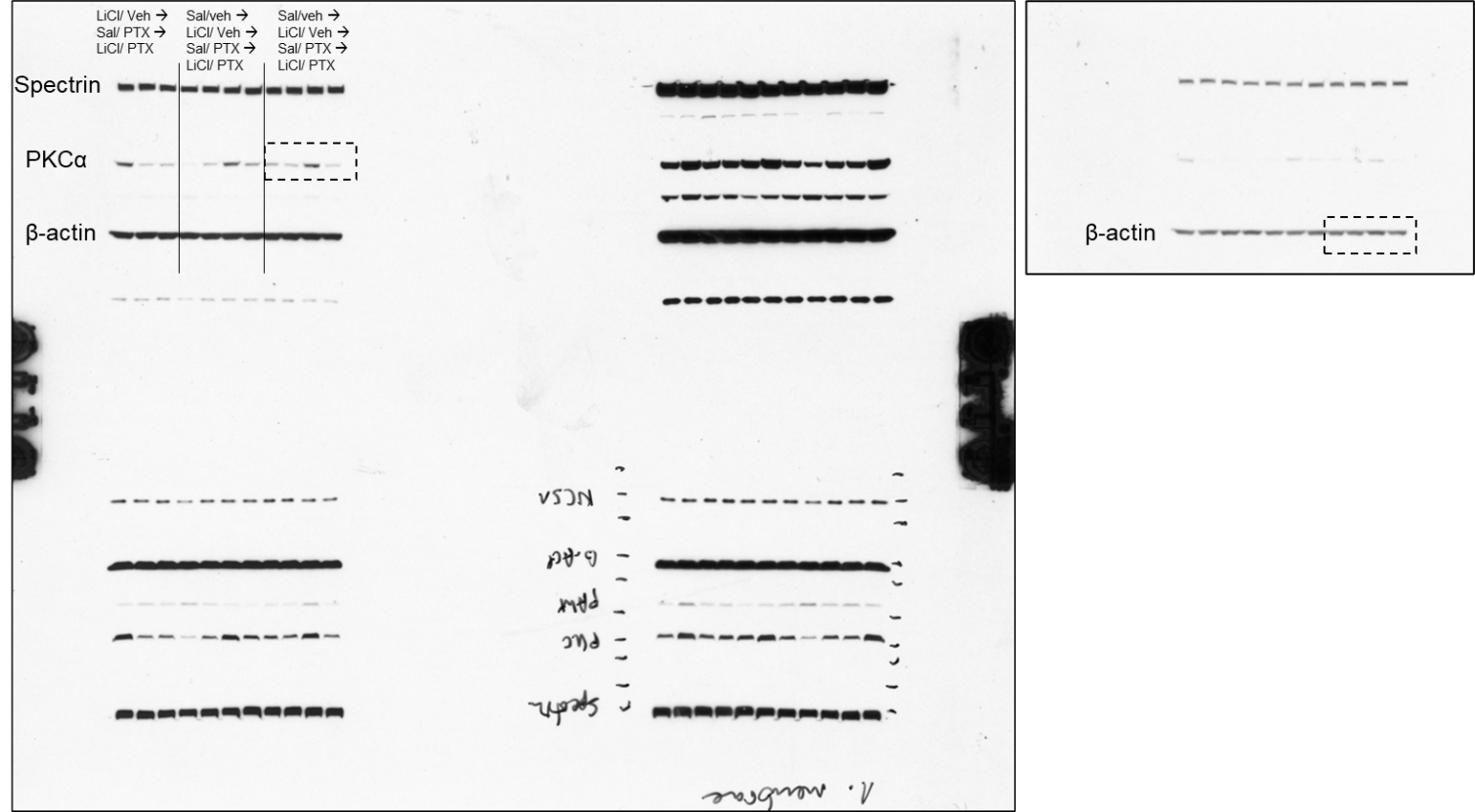
Ref. Fig. 5A: 30 DPI cortex PKCα & β-actin

Ref. Fig. 5B: 30 DPI hippocampus PKCα & β-actin
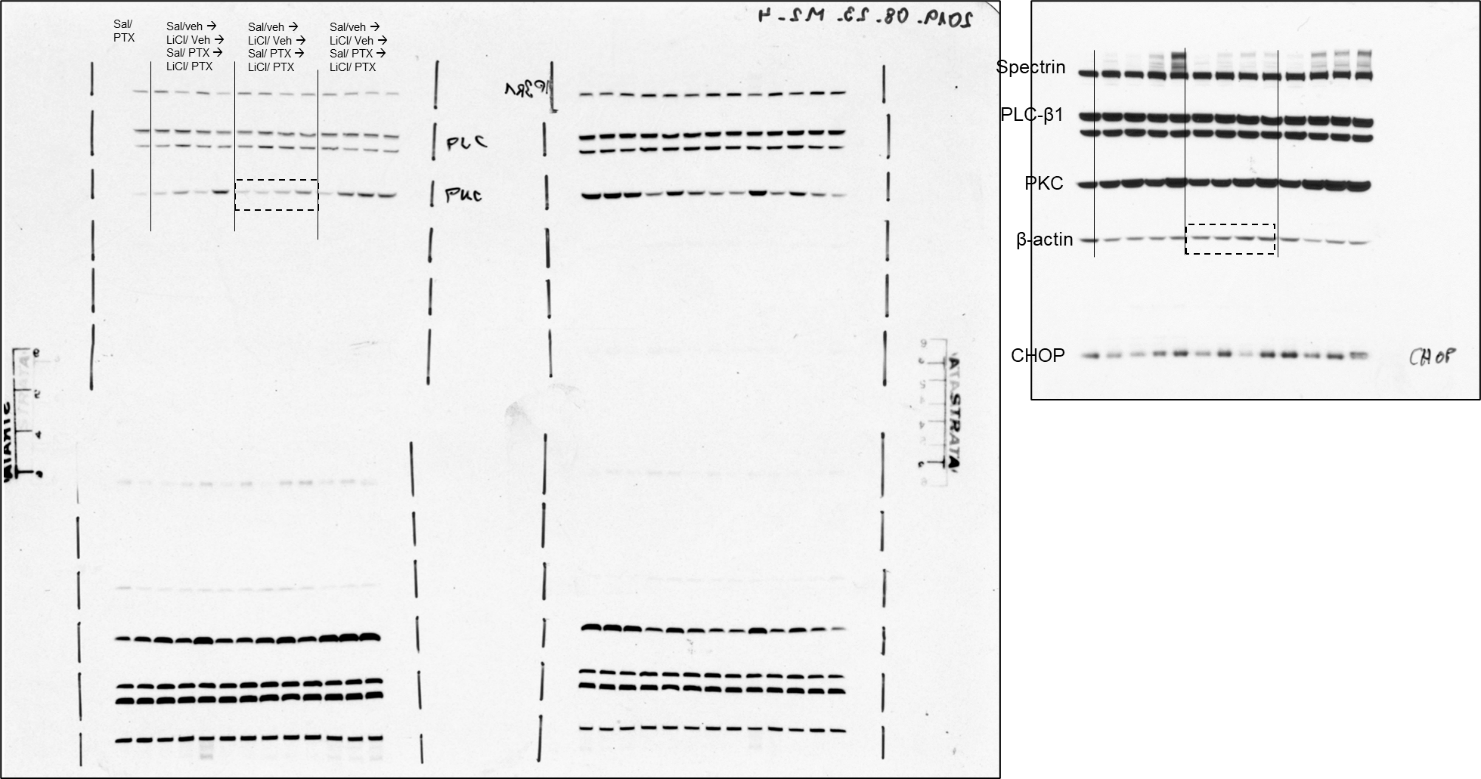


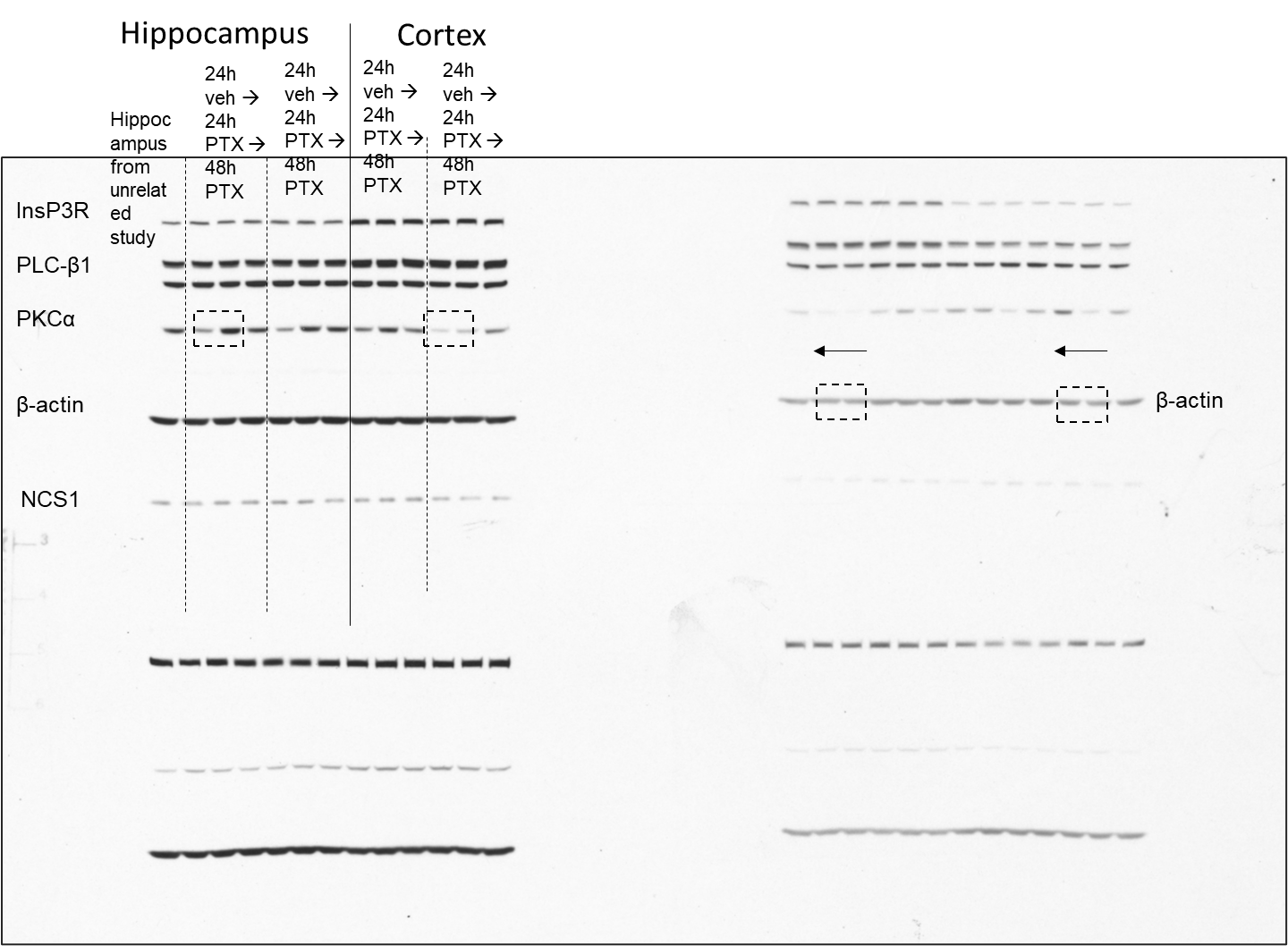
Ref. Fig. 5C and D: Acute hippocampus and cortex PKCα & β-actin

Ref. Fig. 5E and 5F: Acute hippocampus and cortex p-MARCKS and t-MARCKS


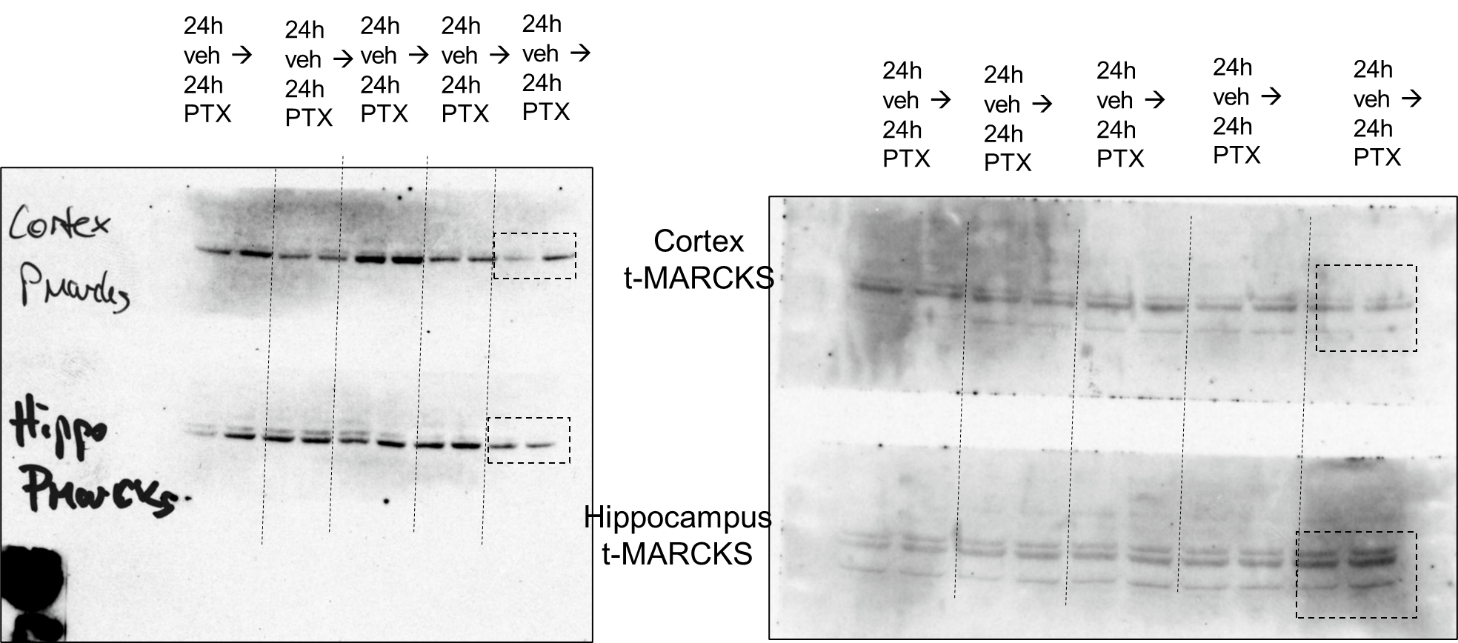

Supplement: Supplementary file 9 — Additional file 9 Original files for traced images [file 13024_2021_463_MOESM9_ESM.docx]
